# Strategy Switching in the Stabilization of Unstable Dynamics

**DOI:** 10.1371/journal.pone.0099087

**Published:** 2014-06-12

**Authors:** Jacopo Zenzeri, Dalia De Santis, Pietro Morasso

**Affiliations:** Robotics, Brain and Cognitive Sciences Department, Istituto Italiano di Tecnologia, Genoa, Italy; University of Münster, Germany

## Abstract

In order to understand mechanisms of strategy switching in the stabilization of unstable dynamics, this work investigates how human subjects learn to become skilled users of an underactuated bimanual tool in an unstable environment. The tool, which consists of a mass and two hand-held non-linear springs, is affected by a saddle-like force-field. The non-linearity of the springs allows the users to determine size and orientation of the tool stiffness ellipse, by using different patterns of bimanual coordination: minimal stiffness occurs when the two spring terminals are aligned and stiffness size grows by stretching them apart. Tool parameters were set such that minimal stiffness is insufficient to provide stable equilibrium whereas asymptotic stability can be achieved with sufficient stretching, although at the expense of greater effort. As a consequence, tool users have two possible strategies for stabilizing the mass in different regions of the workspace: 1) high stiffness feedforward strategy, aiming at asymptotic stability and 2) low stiffness positional feedback strategy aiming at bounded stability. The tool was simulated by a bimanual haptic robot with direct torque control of the motors. In a previous study we analyzed the behavior of naïve users and we found that they spontaneously clustered into two groups of approximately equal size. In this study we trained subjects to become expert users of both strategies in a discrete reaching task. Then we tested generalization capabilities and mechanism of strategy-switching by means of stabilization tasks which consist of tracking moving targets in the workspace. The uniqueness of the experimental setup is that it addresses the general problem of strategy-switching in an unstable environment, suggesting that complex behaviors cannot be explained in terms of a global optimization criterion but rather require the ability to switch between different sub-optimal mechanisms.

## Introduction

Using a tool or shaping a new tool for solving a task are important aspects of sub-symbolic human cognition, but this is not a prerogative of humans: primates [1], [2], [3] and even crows [4] can exhibit such skilled behavior. Moreover, it has been demonstrated that the skilled use of tools implies a modification of the body schema that incorporates the tool as a functional extension of the body [5], [6].

If a task is stable and the tool is sufficiently rigid, the incorporation of the tool in the body schema and its control require a rather straightforward reorganization of the coordination patterns, equivalent to the modification of the Jacobian matrix of the end-effector. On the contrary, compliant tools employed in unstable tasks require learning of a novel control mechanism by careful integration of multi-joint/multi-limb coordination with stabilization. An example of an underactuated compliant tool is a fishing rod, which is characterized by an infinite number of uncontrollable degrees of freedom. The arm-rod system is described as underactuated because the configuration of the rod is only partially affected by the motor control patterns of the arm but is mainly determined by its intrinsic dynamics. On the other hand, unstable tasks are common components of human activities, like screwing/unscrewing, drilling, inserting a peg in a hole, chiselling, manipulating soft tissues (like in surgery), balancing a pole etc. This study addresses both issues (underactuated control and unstable task) at the same time, further developing a previous work [7] in which naïve subjects were exposed to the unknown tool in an unknown environment and managed to find out suitable stabilization strategies.

The tool, which is simulated by a bimanual haptic robot with direct torque control of the motors, consists of a mass (the ‘tool-tip’) and two hand-held non-linear springs. The tool-tip operates in an unstable environment, characterized by a saddle-like force-field. The non-linearity of the two springs allows users to affect size and orientation of the tool stiffness ellipse, by using different patterns of bimanual coordination of the two spring terminals: minimal stiffness occurs when the two terminals are aligned and grows when they are stretched apart. The tool parameters are set such that minimal stiffness is insufficient to provide stable equilibrium of the tool-tip. However, asymptotic stability can be achieved with sufficient stretching at the expense of greater effort. As a consequence, tool users have two possible strategies for stabilizing the tool-tip in different regions of the workspace: 1) high stiffness strategy (Stiffness Stabilization Strategy - SSS), aiming at asymptotic stability, and 2) low stiffness positional strategy (Positional Stabilization Strategy - PSS), aiming at bounded stability in analogy with manual stabilization of an inverted pendulum.

Unstable tasks have been studied by different authors but these previous experimental designs did not allow for the use of more than one strategy. Consider, for example, the inverted pendulum balancing task by means of a manually operated spring whose stiffness is smaller than the rate of growth of the destabilizing gravity-related torque [8]: in this case, the SSS is infeasible, although it is still possible for subjects to succeed at the task. Indeed, they solve the task by producing intermittent stabilization gestures that achieve a regime of bounded oscillations (PSS). Moreover, by decreasing the moment of inertia of the system while keeping constant all the other parameters, the authors observed increased oscillations and we may expect a bifurcation point beyond which subjects would fail the stabilization task. The bifurcation point can be characterized by the falling time constant of the system or the length of an equivalent inverted pendulum: if the time constant is too small (less than 100 ms) or the equivalent inverted pendulum is too short (less than 10 cm), stabilization cannot be achieved. In other words, the PSS has a limited dynamic range, determined by biological parameters, such as the delay of the sensory feedback. Another example, which illustrates an opposite stabilization strategy, is given by the divergent force-field compensation experiments [9], [10]: in this case, the PSS is infeasible because the falling time constant determined by the system parameters is too small. Nevertheless, subjects can learn muscle co-activation patterns that modulate size and orientation of the hand stiffness ellipse in an optimal way, i.e. align the major axis of the ellipse with the direction of the divergent field. In other words, in this example the subjects are forced to choose the SSS due to the parameters of the designed system and muscle elastic properties allow them to achieve the goal. Similar observations apply both to the pole balancing task, because the stiffness is null by construction without any possibilities to increase it, and to human upright standing, because the ankle stiffness is smaller than the critical level determined by gravity [11], [12]. It is worth mentioning that, in the upright standing paradigm, the compliance of the Achilles tendon prevails [13] making co-activation of the ankle muscles ineffective for modulating the ankle stiffness. This does not occur in the case of the arm neuromuscular system because the stiffness of the tendons is great enough for modulating effectively the stiffness ellipse through the co-activation of antagonist muscles.

The virtual tool described in this paper was designed with the goal of allowing users to choose the stabilization strategy. It is a novel design and, although it is somehow artificial, we think it matches aspects of skilled behavior in real life situations, and thus, can shed some light on how humans deal with problems where multiple strategies are possible.

The stiffness stabilization strategy (SSS) is basically open-loop and is characterized by a large frequency band, because the implicit positional feedback comes from the elastic properties of the muscles and/or the tool is instantaneous. On the other hand, this strategy requires a high effort level because the stiffness grows with increasing effort [14], [15], [16], [17] and, for the same load, increasing the stiffness requires some kind of co-activation of antagonist elements [18], [19]. In this way it is possible to achieve an ‘asymptotic’ stability and thus residual oscillations around equilibrium can be attributed to motor noise. The latter grows increasing effort [20], [21], but can be reduced by impedance control [22].

The positional stabilization strategy (PSS) has a smaller bandwidth, because it employs delay-affected explicit positional feedback, but is characterized by a lower level of effort, because it does not require co-activation of antagonistic elements. Following this strategy, the feedback loop can be operated in continuous or discontinuous time. In the former case, however, the large delay in the control loop strongly limits the range of the controller's parameter values that can provide static stability (i.e. compensate the unstable load) without inducing dynamic instability. Intermittent control is more robust [23], [24], [25] provided that the dynamics of instability are not too fast, i.e. the time constant of the dominant unstable pole is not too small. An intermittent, closed-loop controller of an unstable load, operating with a low level of stiffness, can avoid instability achieving ‘bounded stability’. In this case, the residual oscillations around equilibrium are mainly determined by a discrete train of event-driven, predictive stabilization bursts rather than intrinsic motor noise.

In summary, the two stabilization strategies are characterized by a trade-off between effort and frequency band: if a subject aims at utilizing a small effort, he must accept a small bandwidth, whereas if he wishes or is forced to have a high bandwidth, he must be prepared to apply a suitable amount of effort. However, the choice between the two strategies is not granted in general since the feasibility of either strategy depends on the ‘ergonomic design’ of the arm+tool system.

In the aforementioned paper [7], naïve subjects were asked to stabilize the end-point of the virtual tool in different target areas of the workspace, where a saddle-like force-field was active. The task had two implicit components: 1) an ‘equilibrium sub-task’, which required the two hands to transmit a pair of force vectors to the end-point whose vectorial composition compensated the local force field; 2) a ‘stabilization sub-task’, aimed at counteracting the instability of the field using one of the two strategies defined above. In the SSS the two sub-tasks coincide because the achieved equilibrium is stable, whereas in the PSS they are independent. We wish to emphasize the fact that the task is strongly space-variant because the coordination/stabilization patterns vary widely in different parts of the workspace. After an initial exploration of the dynamics of the system, facilitated by the fact that the force-field remained permanently active, all naïve subjects succeeded in solving the task in the time course of a single session. They did so by quickly settling for one of the two strategies and keeping it consistent for the rest of the experiment: about 60% of the subjects adopted the SSS and 40% the PSS. The fact that the effort required in the former case is about twice the effort in the latter, suggests that minimizing effort is not a primary concern. On one hand, the subjects who adopted the SSS learned a bimanual coordination that oriented the stiffness ellipse of the virtual tool in the optimal way, i.e. they learned to separate the two handles of the virtual tool in the medio-lateral direction. On the other hand, the subjects who adopted the PSS kept the two handles very close to each other, minimizing effort.

In this paper, using the same experimental setup as described in the previous study, we report the results of a study in which subjects were trained to become ‘conscious’ expert users of the virtual tool in both strategies. After they became proficient, we conducted a preliminary study to test their generalization capabilities. In this second experiment, we assessed their performance with the same virtual tool in a continuous tracking task that criss-crossed the target areas of the former one. This experiment allows us to observe whenever the switching mechanism between the two strategies occurs, from the high-stiffness to the low-stiffness regime and back.

## Materials and Methods

### The Virtual Tool

**Figure 1:**
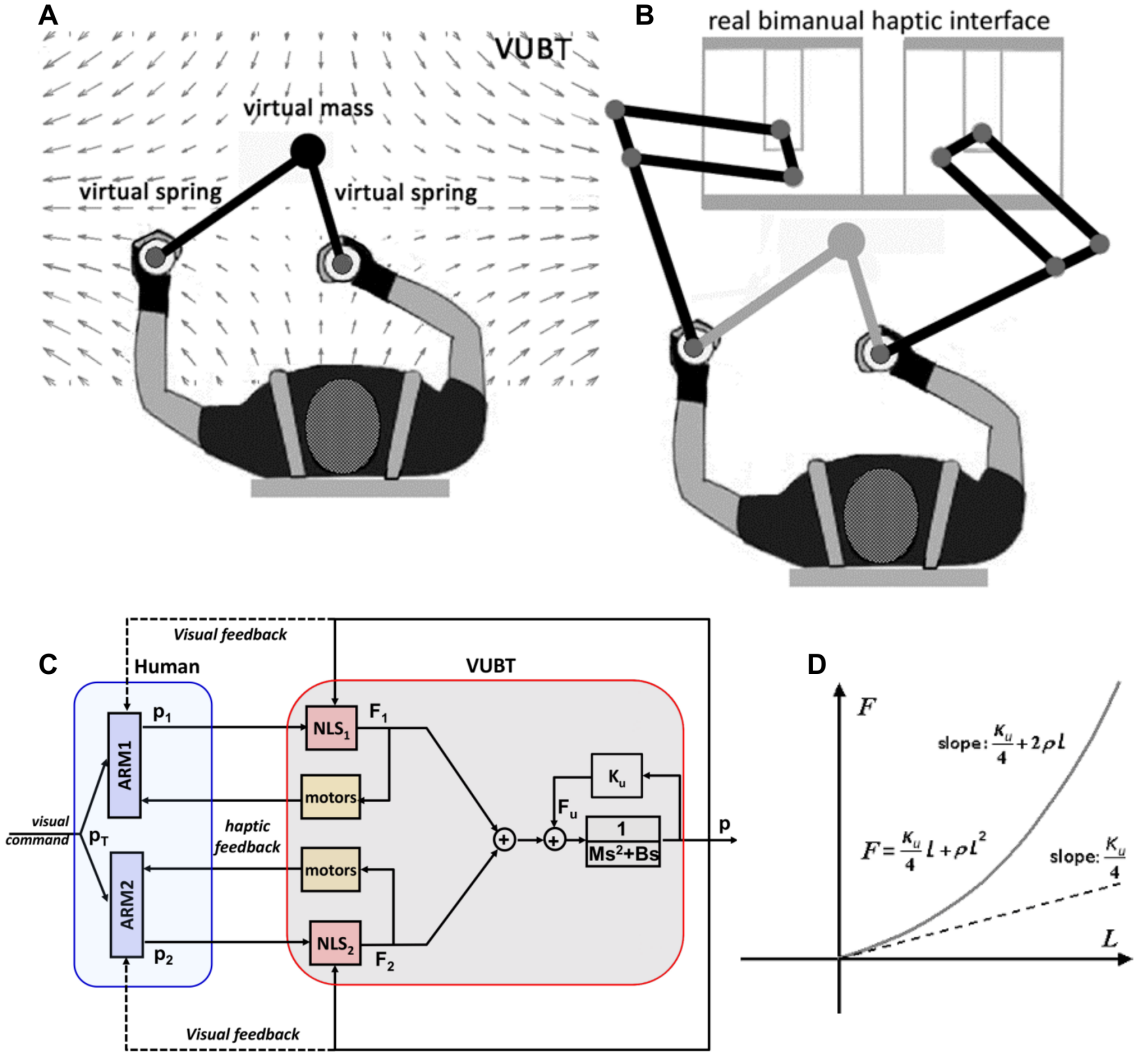
Features of the Virtual Underactuated Bimanual Tool (VUBT). **Panel A:** Sketch of the VUBT, which includes a virtual mass and two non-linear virtual springs; a saddle-like force-field is applied to the virtual mass, making the task unstable. **Panel B:** Implementation of the VUBT by means of a bimanual haptic interface. **Panel C:** Block diagram of the VUBT, interacting with the human user. **Panel D:** Length-tension curve of each NLS (non-linear, virtual spring).

The subjects were trained to use a Virtual Underactuated Bimanual Tool (VUBT, Figure 1). VUBT consists of two elastic linkages, or virtual springs, connected to a virtual point mass, which is the end-point of the virtual tool or tool-tip. The free-ends of the two springs are grasped by the subjects and thus, for each time instant, the shape of the VUBT is a triangle. The general task is to indirectly control the position of the tool-tip 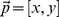 in order to reach or track a target 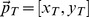 in the workspace by acting on the position of the two spring terminals (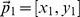 and 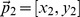). The positions of the target, the tool-tip, and the terminals of the two springs are visualized on a screen.

The tool-tip has a virtual mass *M* and is under the action of the destabilizing force 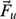, generated by a saddle-like force-field, and the two elastic forces, (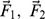), determined by the two non-linear springs. The user can control amplitude and direction of the two forces by choosing the position of the two terminals in relation to the current position of the tool-tip. The overall dynamics of the virtual tool is then characterized by the following equation, where 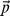 is the controlled variable and 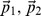 are the two control variables:
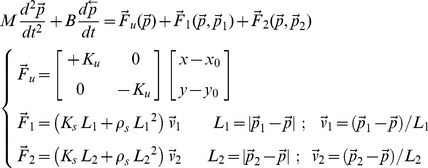
(1)


The origin of the saddle field [*x_0_,y_0_*] is on the sagittal plane of the subject, about 0.5 m forward, with respect to the chest; the *x*-axis is aligned medio-laterally and is the unstable manifold of the field; the *y*-axis is aligned in a posterior-anterior way and is the stable manifold of the field. A viscous field (characterized by the parameter *B*) carries out a damping action. *L_1_, L_2_* are the lengths of the two springs; *K_s_, ρ_s_* are the spring parameters. VUBT is ‘underactuated’ because it is impossible to simultaneously control the position of the end-point and the angle between the two linkages: such an angle is an internal degree of freedom that is not directly controlled by the hands but depends on the interaction with the environment.

The stiffness of each elastic linkage is not constant but grows linearly with the degree of stretch:
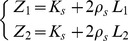
(2)


From this, we can derive the overall stiffness matrix of VUBT, which characterizes the interaction between the virtual mass and the environment:
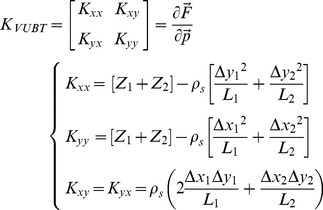
(3)where 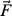 is the total active force applied to the virtual mass; Δ*x_1_  =  x_1_–x*; Δ*y_1_  =  y_1_–y*; Δ*x_2_  =  x_2_–x*; Δ*y_2_  =  y_2_–y*. This gives the explicit dependence of the four elements of the matrix from the coefficients of elasticity (*K*_s_, *ρ*_s_) and the positions of the two hands with respect to the tool-tip. Therefore, the subject can indirectly determine the size and orientation of the stiffness ellipse of the tool in order to achieve equilibrium and/or stability.

The element of the stiffness matrix that is most relevant from the point of view of stability is *K_xx_* because it subtracts its value from the instability coefficient *K_u_*, reducing the degree of instability along the medio-lateral unstable manifold. This consideration is apparent if we linearize the nonlinear dynamics of the virtual tool (equation 1) in the neighborhood [δ*x*, δ*y*] of an equilibrium state [*x_e_*, *y_e_*]:
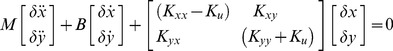
(4)


A necessary and sufficient condition for the asymptotic stability of the equilibrium point is that the eigenvalues of the elastic matrix are both negative and this implies, in particular, that 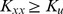. From this, we can define a condition of ‘marginal (asymptotic) stability’ (*K_xx_*  =  *K_u_*) and a corresponding indicator, namely *SSI* (Stiffness Size Index):
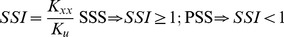
(5)


Supposing that both springs are stretched by an equal amount *L* (*Z_1_  =  Z_2_  =  Z  =  K_s_ + 2ρ_s_L*), *K_xx_* has a range of possible values between a maximum of *K_xx_  =  2Z*, when both springs are aligned with the *x*-axis, and a minimum of *K_xx_  =  2Z – 2ρ_s_L*, when both springs are aligned with the *y*-axis. If *L* = 0, i.e. if the two hand positions coincide, the stiffness ellipse has the minimum size and becomes a circle of radius 2*K*_s_. In any case, the ellipse size increases with the stretching of the two springs and its major axis is approximately aligned with the inter-hand segment. Therefore, the overall stiffness of the tool+arms system can be increased/decreased by coordinating the motion of the two hands, with respect to the tip of the virtual tool, in such a way as to stretch/un-stretch the two springs.

### Configuration of the Virtual Tool

The system's parameters were chosen in such a way as to make both strategies possible for a human subject, which means that the following conditions are satisfied: 1) the minimum value of the overall stiffness, which occurs when both springs are completely unloaded, is less than the instability coefficient of the field, in order to yield a chance to the PSS; 2) in that condition, the unstable time constant is long enough to make the PSS feasible; 3) the degree of stretching of the elastic linkages, which is necessary for making the SSS effective, is such that the required effort is within the human capability, although fatiguing. More specifically the following selections were made:

1. In the workspace of the experiments (a circle with a radius of 10 cm), there should be a range of forces that is challenging and fatiguing but not too much, i.e. it should be sustained by normal subjects for the duration of a session (one hour or more). From this, we derived the value of the force-field coefficient: *K*_u_ = 592 N/m. In this way, the range of forces that will be experienced by the subjects does not exceed 50 N.
2. The other parameters were calculated by considering the intrinsic dynamics of the virtual mass *M* along the stable and unstable manifolds of the force-field, in the absence of control actions:
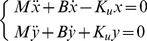
(6)The motion along the stable manifold is a damped oscillation with natural frequency ω_n_ and damping factor ζ. The motion along the unstable manifold is characterized by two exponentials, one with a negative time constant and the other with a positive (unstable) time constant τ_u_. These three coefficients are related to the parameters of the virtual tool by the following equations:
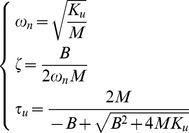
(7)
  Having already selected *K_u_* we still need to choose *B* and *M* in such a way that 1) the ‘falling time constant’ τ_u_ is sufficiently long, e.g. comparable to the falling time constant of the human standing body (about 0.3 s), 2) the natural frequency is not too high (of the order of 1 Hz), and 3) the damping ratio is not too small. A good trade-off among the three requirements is provided by the following choice of parameters:
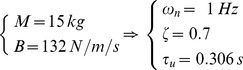
(8)
3. For the selection of the spring parameters (*K_s_*, ρ_s_) the following criteria were used: 1) in the center of the force-field, with unloaded springs (L_1_  =  L_2_ = 0), the stiffness of the virtual manipulandum along the unstable manifold should be only a fraction (e.g. ½) of the value required for marginal stability: *K_xx_*  =  2*K_s_*  =  0.5*K_u_* → *K_s_* = 148 N/m; 2) for the selection of ρ_s_ we can assume that, if the virtual mass is in the center of field, marginal stability could be obtained by aligning the two springs on the *x*-axis and stretching each of them by *L* = 0.05 m. From this we get ρ_s_ = 1480 N/m^2^.

Summing up, the set of parameters that allows operating with similar ease in both stabilization strategies is listed in table 1.

**Table 1:** Parameters of the VUBT.

M	15 kg
B	132 N/m/s
K_u_	592 N/m
K_s_ = K_u_/4	148 N/m
ρ_s_	1480 N/m^2^

### Sensitivity analysis

Table 1 reports the VUBT parameters that, in theory, could allow a subject to choose either strategy for stabilizing the end-point of the virtual tool in the unstable field in different positions of the workspace. The experiments performed with naïve subjects [7] show that this freedom of choice is real. However, it remains to be seen how sensitive the actual behavior of the subjects is to variations of the system parameters. In this framework, we may expect that the two strategies have different limiting factors.

1. Limiting factors for the PSS. The critical coefficient, in this case, is the value of the falling time constant τ_u_. The relationship between τ_u_ and *M*, for a given ζ, is the following one:
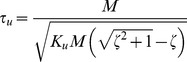
(9)By reducing *M* (and *B* accordingly with the dynamics of the system) while keeping invariant the damping factor ζ, the time constant becomes smaller and smaller, making the task of balancing the load by trains of corrective commands more and more difficult. For example, for *M* = 1 kg τ_u_ = 0.079 s and in this operating region, we expect that even an experienced user would be unable to use the PSS.
2. Limiting factors for the SSS. The critical coefficient, in this case, is the damping factor ζ. The stiffness strategy is effective in achieving asymptotic stability but if the damping is too low, it is difficult to stabilize the mass in the target area. For the PSS, on the contrary, there is no difference between compensating for the oscillations due to underdamping or to instability, provided that the falling time constant is not too short. For a given value of τ_u_, *M* and *B* vary as a function of the damping factor according to the following equations:
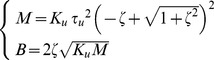
(10)

For example, equation 10 implies that if ζ is decreased from 0.7 to 0.1, *M* must be increased from 15 kg to 45 kg and *B* must be decreased from 132 N/m/s to 33 N/m/s.

### Equilibrium & Stability Conditions

Given a target point for the virtual mass 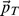 in the workspace, equilibrium is characterized by the following equation:
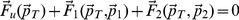
(11)


**Figure 2:**
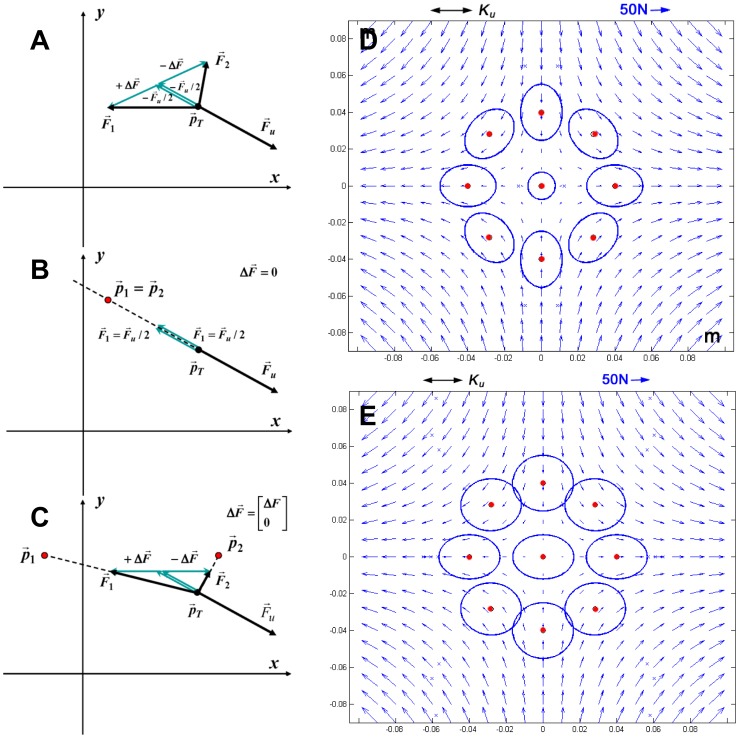
Equilibrium conditions of the virtual tool in a given target point. 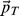
 **Panel A:** The local field vector 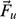
can be equilibrated by the two non-linear springs in infinite number of ways which can be expressed by adding to the balanced, minimum-effort solution (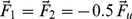) a pair of opposite forces (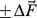) of arbitrary magnitude and orientation. **Panel B:** Equilibrium condition in the PSS, characterized by 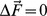; **Panel C:** Equilibrium condition in the SSS, characterized by an additional stretch 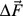, with amplitude sufficient to achieve marginal stability, i.e. *K_xx_  =  K_u_*; **Panel D:** The stiffness ellipses of the PSS are aligned with the local orientation of the field. **Panel E:** The stiffness ellipses in the SSS are oriented medio-laterally, i.e. are aligned along the unstable manifold of the field.

The control variables are the positions of the two elastic linkages: 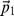 and 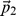. Therefore, the equilibrium is redundant in the sense that there are an infinite number of solutions that satisfy equation 11. As illustrated in Figure 2 (panel A), such infinite set of solutions is obtained by adding, to the balanced solution (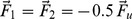), a pair of opposite forces (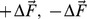) of arbitrary magnitude and orientation.

Such double infinite set of solutions differs for two behaviorally relevant features: 1) the required effort and 2) stability. With regards to the former point, it is easy to demonstrate that the ‘minimum-effort solution’ is characterized by the fact that the two hands are superimposed and aligned with the direction of the force-field:
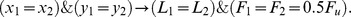



We may name this solution *standard bimanual coordination* pattern, which describes the PSS well. After learning this strategy, the subjects implicitly carry out the following computational scheme (see also Figure 2, panel B):

1. sense intensity *F_u_* and direction 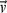 of the local force-field, for a given target point 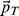;
2. compute the stretch δ to apply to both springs: 
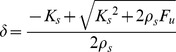
;
3. position both hands accordingly: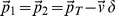.

By adopting this strategy, a user would handle the tool in such a way as to exhibit stiffness ellipses that are oriented as the local force-field (Figure 2, panel D). However, if the target point is not too far from the origin of the field, equilibrium is not asymptotically stable because *K_xx_ < K_u_*.

When learning the SSS, subjects quickly understand that the solution of minimum effort implies a medio-lateral separation of the two hands, thus inducing a pair of opposite forces (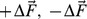) aligned with the *x*-axis. Then they need to learn the amplitude of such forces, such that the condition of asymptotic stability is satisfied: *K_xx_ ≥ K_u_*. Thus, after learning this strategy, the subjects implicitly carry out the following computational scheme (see also Figure 2, panel C):

1. sense intensity and direction of the local force-field 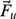;
2. choose a pair of forces 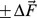 aligned with the *x*-axis;
3. compute the overall forces that must stretch the springs 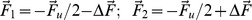;
4. compute the amount of stretch of the springs 
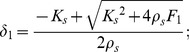
 
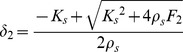
and the line of action of the two forces 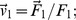 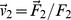;
5. compute the position of the two hands 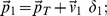 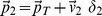 and the critical element of the stiffness matrix 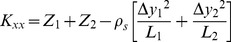;
6. if *K_xx_ < K_u_* increase the amplitude of 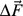 and go to step 3, else terminate the procedure.

By adopting this strategy, a user would handle the tool in such a way as to exhibit stiffness ellipses that are aligned with the *x*-axis (Figure 2, panel E).

**Figure 3:**
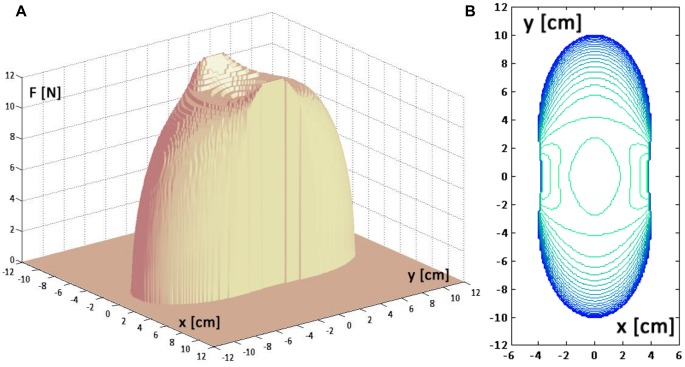
Forces and stability. Distribution over the workspace of the magnitude of the pair of 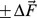 forces that must be applied in order to reach the condition of marginal stability. **Panel A:** 3D-plot; **Panel B:** contour plot. The *x*-axis corresponds to the medio-lateral direction (the unstable manifold of the force-field); the *y*-axis corresponds to the antero-posterior direction (the stable manifold).

We applied this procedure to all the points of the workspace, in order to understand the distribution of the additional pair of forces for marginal stability and the nature of the task that must be solved by the users (see Figure 3). It appears that the amplitude of such pair of forces remains approximately stationary in the neighborhood of the origin (with a value of about 12 N). Then it decreases smoothly in the *y*-direction (within a range of about ±10 cm) and much more abruptly in the *x*-direction (with a smaller range of about ±4 cm). The locus of the positions at which the amplitude of 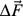 becomes null, i.e. the locus of marginal stability, is ‘peanut-shaped’ and is more elongated in the *y-* than in the *x*-direction. Let us further clarify the meaning of Figure 3. When a subject attempts to stabilize the end-point of the virtual tool in the center of the workspace according to the SSS, the figure says that the minimum amount of medio-lateral separation of the two hands corresponds to a force 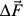  = 12 N. The figure says also that such a minimum degree of separation decreases quickly if the end-point is moved laterally and more slowly for forward/backward movements. Ultimately, the degree of separation and the corresponding force 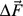 become null for a lateral displacement from the center of the workspace greater than ±4 cm or for a forward/backward displacement greater than ±10 cm. For larger displacements and, more generally, for equilibrium points outside the peanut-shaped contour, the distinction between SSS and PSS disappears: there is no need to stretch laterally the two virtual springs and asymptotic stability is achieved anyway.

From equation 3 we learn that the smallest possible value of *K_xx_* is achieved when the two springs are unloaded: *L_1_ = L_2_* = 0 → *K_xx_*  =  0.5*K_u_*. In the standard bimanual coordination, this equilibrium condition is characterized by null effort and can only occur when the target is in the origin of the force-field. It is also unstable because *K_xx_* is only half the minimum value required by marginal stability. For target areas which are situated increasingly away from the origin, the field intensity (as well as the minimum effort) grows linearly with the distance. As a consequence, also the corresponding VUBT stiffness increases, and in particular the critical *K_xx_* element, gradually approaching the condition of marginal stability defined above. Figure 3 includes the peanut-shaped locus of points for which marginal stability is achieved in the case of standard bimanual coordination:

• For the points inside the locus the subjects have a choice: 1) to stick to the standard coordination, which minimizes the effort, forcing them to achieve bounded stability in an active way by means of carefully timed trains of corrective commands; 2) to supplement the standard coordination by stretching both springs up to the point in which asymptotic stability is achieved with a suitable pair of forces 
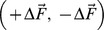
, at the expense of increased effort. In this case, the subjects can also choose the direction in which to apply the opposite forces 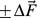. This direction affects the orientation of the stiffness ellipse in a rather complicated way. However, it is possible to demonstrate numerically that if Δ*F* is comparable to the intensity of the local force field the orientation of the stiffness ellipse is very close to the orientation of 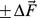. Thus, in the optimal stiffness strategy the 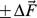 is oriented along the unstable manifold of the field, i.e. medio-laterally. Instead, the amount of lateral stretching is not unique as it depends on the distance from the origin and the position of the target. Figure 3 shows that the required stretch is a decreasing function of the distance from the origin. Therefore, a subject that has learnt to stabilize the virtual mass in the center (according to the stiffness strategy) will be able to apply the required lateral stretch also in the periphery of the workspace.

• For target points outside that locus, the standard coordination pattern satisfies both the equilibrium condition (equation 11) and the stability condition at the same time.

During transient corrective motions, the equilibrium condition expressed in equation 11 is not satisfied and the following equation holds: 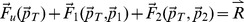



Therefore, a pair of forces 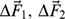 exists such that the resultant of the forces 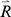 acting on the virtual mass is not null and motion occurs. We can distinguish two possible situations: 1) in the case of the SSS, 
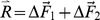
 and 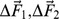 can have arbitrary magnitude and orientation, but, as is characteristic of the strategy, opposite direction; 2) in the case of the PSS, the two hands are overlapped and consequently 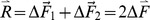. 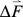 can have arbitrary magnitude and orientation. If the angle between 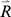 and 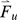 is small, we can assume that the dynamics of the system is dominated by the unstable force-field. Otherwise, we can assume that a corrective motion takes place.

### Implementation of the virtual tool

The virtual tool described in the previous section was simulated by means of a bimanual haptic interface (BdF2, Celin srl, La Spezia, Italy), which is a direct bimanual evolution of the planar robot ‘Braccio di Ferro’ [26]. Each robot has a large planar workspace (0.8×0.4 m ellipse) and a rigid structure with two direct-drive, brushless motors, and low intrinsic mechanical impedance. These features allow direct estimation of forces applied to the hand of the user by the robot from the commanded currents. Each robot can measure the trajectory of the hand with high-resolution (0.1 mm) and is capable of applying forces at the corresponding handle. The control architecture is based on the real-time operating system RT-Lab® and includes three nested control loops: 1) an inner 16 kHz current loop; 2) an intermediate 1 kHz impedance control loop; 3) an outer 100 Hz loop for visual display and data storage. The two identical planar robots are mounted in a mirrored configuration on the same rigid frame, which allows independent regulation of vertical and horizontal position. They are positioned horizontally as close as possible (distance between the axes of the motors 38.5 cm), in order to maximize the overlap of the corresponding workspaces. The vertical position of the robot linked to the right hand was adjusted for each subject in order to keep the right arm approximately horizontal. The other robot was slightly shifted downward in order to avoid interference between the two arms. For the same reason, the handle of the former robot was directed upward, whereas the handle of the latter robot was directed downward. The positions of the two handles were calibrated with respect to a common reference frame, which was used for all the relevant variables of the experiments. The robot is equipped with a 24.5″ LCD screen with resolution of 1920×1200 and 100 Hz refresh rate. The workspace is mapped on the screen with a unary magnification factor so that a displacement of 1 cm of the hand corresponds exactly to 1 cm on the screen. The screen is positioned vertically in front of the subject and horizontally 30 cm beyond the center of the workspace.

### Subjects, Task & Protocol

Eight healthy, subjects (age  =  29.1±1.6 years) participated in the experiment (Table 2). The research conforms to the ethical standards laid down in the 1964 Declaration of Helsinki that protects research subjects. All the recruited subjects, who were internal doctoral students or post-doc fellows of the Institute, signed a consent form that conforms to the guidelines above. The research also conforms to the protocol “Studio di paradigmi di controllo motorio e adattamento a campi di forza nell'arto superiore mediante utilizzo di interfacce robotiche interattive” (Study of paradigms of motor control and adaptation to force-fields in the upper limb by means of interactive robotic interfaces) approved by the “Comitato Etico” (Ethical Committee) of “ASL 3 Genovese” (the Local Health Authority, which is legally competent for approving experiments involving human subjects).

**Table 2:** Subjects.

Subject	Sex	Age (years)	Height (cm)	Weight (kg)
S1	M	28	178	76
S2	M	29	183	75
S3	M	31	186	70
S4	F	27	171	57
S5	M	28	183	70
S6	F	32	168	58
S7	M	29	175	68
S8	M	29	182	65

Before performing the experiments, the subjects were fully informed about the nature of the task and the existence of two stabilization strategies and were encouraged, during a familiarization phase, to practice both of them. Only after there was evidence that they could indeed switch between one strategy and the other the experiments were started according to the protocol described below.

**Figure 4:**
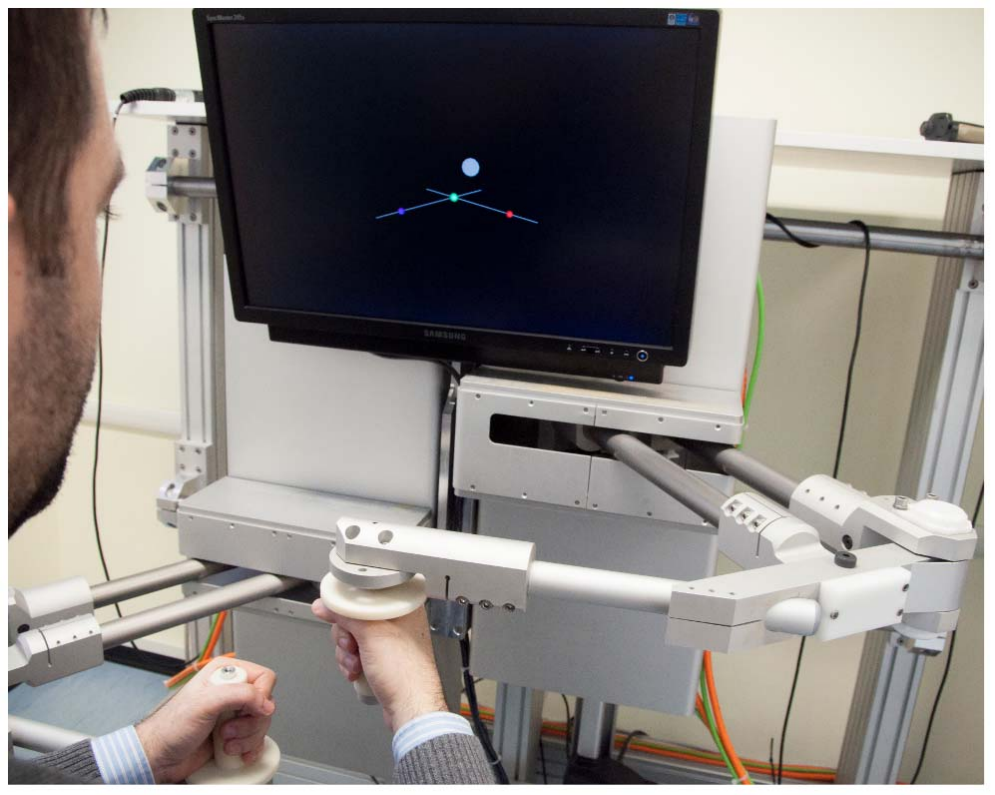
Experimental setup. The subject sits in front of the screen grasping the handles of the bimanual robot with the hands. The screen provides the visual feedback in the following way: the green ball represents the virtual mass, the blue ball the left hand, the red ball the right hand, the gray circle the target position and the two white sticks the virtual springs.

The subjects sat in a chair, with their trunk restrained by means of a seat belt and their sternum aligned with the midline of the bimanual robot at a distance of approximately 40 cm from the center of the workspace. The hands grasped the handles of the robot and faced the screen (Figure 4). Vision of the position of the hand and the robot was not hidden. Nevertheless, subjects were instructed to concentrate on the displayed image and not to look at their hands or the robot's configuration during the experiment.

Two different tasks were used: one task (A) for training the subjects to become experts in the two different stabilization strategies and the other task (B) for a preliminary evaluation of the switching mechanism from one strategy to the other and the generalization capability of the subjects. All the eight subjects took part in task A, while task B was performed by 6 out of the 8 subjects who agreed to participate in the first experiment, since two subjects had to withdraw from the study. The unstable force-field is continuously active throughout the experimental sessions. This is an important difference from other experiments reported in the literature, in which the source of instability is activated/deactivated for each single trial. We believe indeed that robust learning is facilitated by an invariant context.

### Task A

The task is to stabilize the end-point of the virtual tool (represented as a circle with a 1 cm diameter) in one of 9 circular target areas (2 cm diameter), uniformly distributed on the periphery of a circle (8 cm diameter) and in the center. The stabilization requirement is to constrain the tool-tip oscillations inside the current target for a continuous time interval of 4 s. Each experimental session included 2 target-sets (12 center-out movements and 12 return movements per set): the first one performed by using the SSS and the second one by using the PSS. The timing was driven by the behavior of the subjects: the new target was presented after a subject succeeded in stabilizing the mass inside the target region. This means that if a subject entered in the target area but exited before the prescribed time, the time counter was reset for a new stabilization attempt. For this reason, the time required to complete an experimental session varied within 5 minutes (expert user) and 1 hour (naïve user) according to the skillfulness of the subject. We allowed subjects to take a short break of 5 minutes only after completing a target set. The number of sessions was chosen after a pilot test on one subject, who practiced for 22 sessions (44 sets) [27]. Results showed that the subject's performance stabilized after 10 sessions (20 sets) suggesting that a protocol of 10 training sessions might be adequate for our purposes. Therefore, the protocol for task A consisted of 10 sessions on 10 different days, consecutive sessions being no more than 2 days apart.

The values of the dynamical parameters of the system used in the experiment (*M* = 15 kg, *B* = 132 N/m/s) were chosen after evaluating the effect of decreasing the controllability of the tool in every strategy for a single subject. For the PSS, the mass of the tool was systematically decreased while keeping the damping factor ζ constant (this implies to compute the value of B from equation 7) in order to reduce τ_u_. In the case of the SSS, we systematically increased the mass of the system in order to vary ζ, while keeping B constant.

### Task B

**Figure 5:**
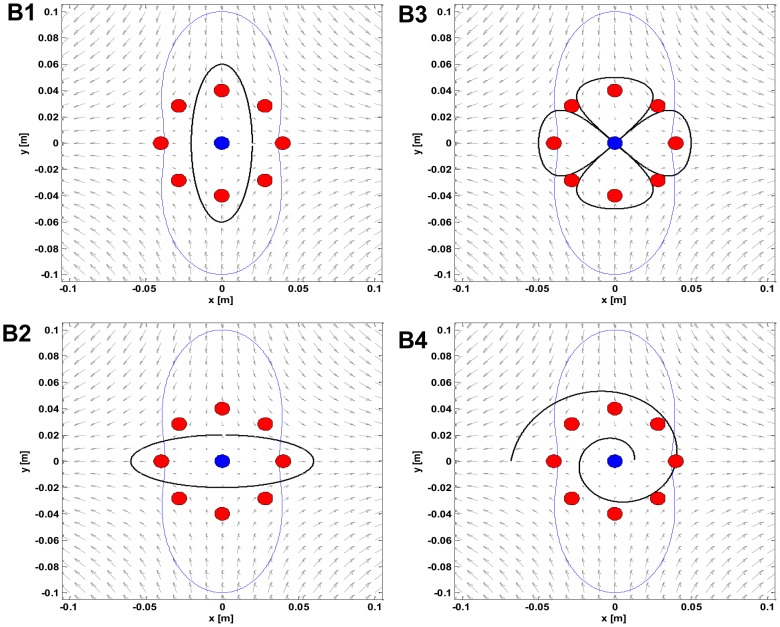
Layout of the targets in the different tasks. In all the panels the eight peripheral targets (in red) and the central parget (in blue) are related to task A. The four panels depict the target trajectories (in black) to be tracked in tasks B. **Panel B1:** Vertical ellipse (swept in the CCW direction). **Panel B2:** Horizontal ellipse (CCW). **Panel B3:** Clover (CW, CCW, CCW, CW). **Panel B4:** Spiral (CCW). In all the panels in the background there is the force-field and the peanut-shaped locus of marginal stability (in blue).

The generalization capabilities of six trained experts (S1, S4, S5, S6, S7, S8) were tested in a single-session tracking exercise along four differently shaped trajectories, as depicted in Figure 5. The goal was to match the motion of a smoothly moving target, generated in the workspace with paths of increasing shape complexity: a vertical ellipse (panel B1) laying in the marginal-stability region; a horizontal ellipse (panel B2) having the extreme points outside the marginal-stability manifold; a clover (panel B3) that combines the two previous shapes; a spiral (panel B4), which stems from the region of marginal stability and ends over the unstable manifold. The subject had no previous knowledge or visual hints about the shape of the path and no constraints regarding the strategy to use to accomplish the task. According to our expectations, however, inside the marginal-stability contour subjects should be free to choose one stabilization strategy or the other. Outside this region, instead, they should be forced to apply the stiffness strategy only.

Each trajectory was presented to every subject four times in a row: the first trial (first lap) served as familiarization with the path and was performed without the unstable force-field, while the following three trials were perturbed by the unstable field. Summarizing, the generalization session is composed of 8 tracking blocks: odd blocks correspond to the familiarization condition and consist of one trial, while even blocks correspond to the unstable condition and last for three trials. The target path was changed every two blocks in the following order: B1 - B2 - B3 - B4. The condition for the beginning of the familiarization trial and the first trial under instability is that the mass is stabilized inside the starting point of the trajectory (diameter = 2 cm) for at least 3 s. In the case of the unstable condition, the subsequent two trials begin without requiring the target to stop on the starting point, so that the tracking continues without interruptions. The laps count follows the target motion irrespectively of the position of the tool-tip, so that every lap is considered complete whenever the target returned in/passed through the starting position. We report the details of the four tracking subtasks hereinafter. On purpose, we chose the velocity of the target to be small enough, in order to present to the subjects a quasi-static stabilization task, although in different parts of the workspace that were not experienced before. For much faster movements the subjects would have to learn a novel and much more difficult task because the stable and unstable manifolds would span a two dimensional plane in a four dimensional space 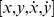, with little chance of generalization from the previous training.

B1) Vertical ellipse

In the subtask B1 the target to be pursued runs along an elliptical trajectory as shown in Figure 5 panel B1 and is described by equation 12:
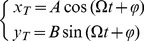
(12)


*A* = 0.02 m and *B* = 0.06 m being the minor and major semi axis of the ellipse respectively; Ω  =  2π/*T* is the angular velocity given *T* = 20 s; *φ*  =  − π. The tracking starts along y in [0.02; 0] m and ends in correspondence to the same point after the completion of one lap (familiarization condition) or three laps (unstable condition) in the counterclockwise direction.

B2) Horizontal ellipse

As for the subtask B2 (Figure 5 panel B2) the ellipse is the same as that in task B1 except being rotated 90° on the x-y plane and takes the following form:
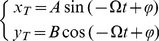
(13)where *A* = 0.02 m and *B* = 0.06 m; Ω = 2π/*T* is the angular velocity given *T* = 20 s; *φ*  =  π. This time, the starting and final points are coincident to [0; 0.02] m. Also in this case, the trajectory is swept in a counterclockwise manner.

B3) Clover

In subtask B3 the target moves along a four-leaved clover shaped trajectory (Figure 5 panel B3), characterized by the following equations:
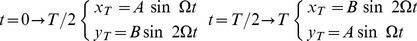
(14)where *A* = 0.05 m; *B* = 0.025 m; Ω  =  2π/*T*, and *T* = 40 s. The initial/final point is the origin [0;0] and every lap starts with the target running along the rightward leaf clockwise (CW), then sweeping the left leaf in a counter-clockwise manner (CCW) and ends after moving from the top leaf to the bottom one. In other words, the first leaf is in the right hemi-space (CW), the second leaf in the left hemi-space (CCW), the third leaf in the upper hemi-space (CCW), and the fourth leaf in the lower hemi-space (CW).

- B4) Spiral

In the last case (subtask B4), the target moved along a spiral trajectory computed according to equation 15:
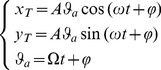
(15)where *A*  =  0.025*/(2π)* m/rad; ω is the angular velocity in rad/s; *φ*  =  π; *ϑ_a_* is the spiral angle with respect to the x-axis and increased linearly with time. In order to limit the increase of task difficulty when moving towards the instability region we computed the angular increment Ω and the angular velocity *ω* as follows:
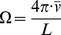
(16)

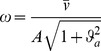
(17)


In equation 16 *L* = 0.3216 m represents the total trajectory length and 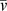  =  0.02 m/s is the desired speed. According to equation 17, ω is computed considering the ratio between the desired speed and the arc length increment in time 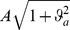. As shown in Figure 5, the spiral is swept in the CCW direction and its starting point is on the x-axis (*x*_T_(0) = 1.25 cm).

### Indicators

For task A, the following indicators were computed during each stabilization interval, as average values in the 4s stabilization time:

- Stiffness Size Index: *SSI  =  K_xx_/K_u_*. It is a dimensionless parameter that identifies the stabilization strategy: SSS (*SSI*>1) or PSS (*SSI*<1). Marginal (asymptotic) stability corresponds to *SSI* = 1.
- Stiffness Orientation Index: *SOI*  =  cos α, where α is the angle between the main axis of the stiffness ellipse and the medio-lateral axis *x*; it identifies the orientation of the stiffness ellipse of the virtual tool.
- Effort Index: *E  =  F_1_ + F_2_* (N). It measures the average total force required for stabilizing the virtual mass.
- Time to Target: *TT* (s). It corresponds to the time interval from the deactivation of the previous target to the last time the tool-tip enters the current target area before its successful stabilization.
- Bimanual Separation Index: 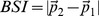 (cm). This index tends to be small in the PSS, with low levels of effort and almost round stiffness ellipses.
- Magnitude of the Velocity Peaks: *MVP* (cm/s). It quantifies the small adjustments during the stabilization in the target areas. It is computed filtering out the velocity peaks with a value less than 80% of the mean velocity adopted by a subject in a particular strategy and peaks that are spaced less than 200 ms apart(5 Hz).
- Frequency of the Velocity Peaks: *FVP* (Hz). It is a measure of the frequency of the corrective bursts in a speed profile during the stabilization phase. It is computed as the reciprocal of the mean temporal difference between consecutive peaks in the speed profile, after filtering out oscillations faster than 5 Hz.

For tasks B we evaluated all the indicators of task A plus the following one:

- Tracking Error: 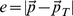 (cm). It quantifies the distance of the virtual mass from the target during the tracking phase.

## Results

### Task A

**Figure 6:**
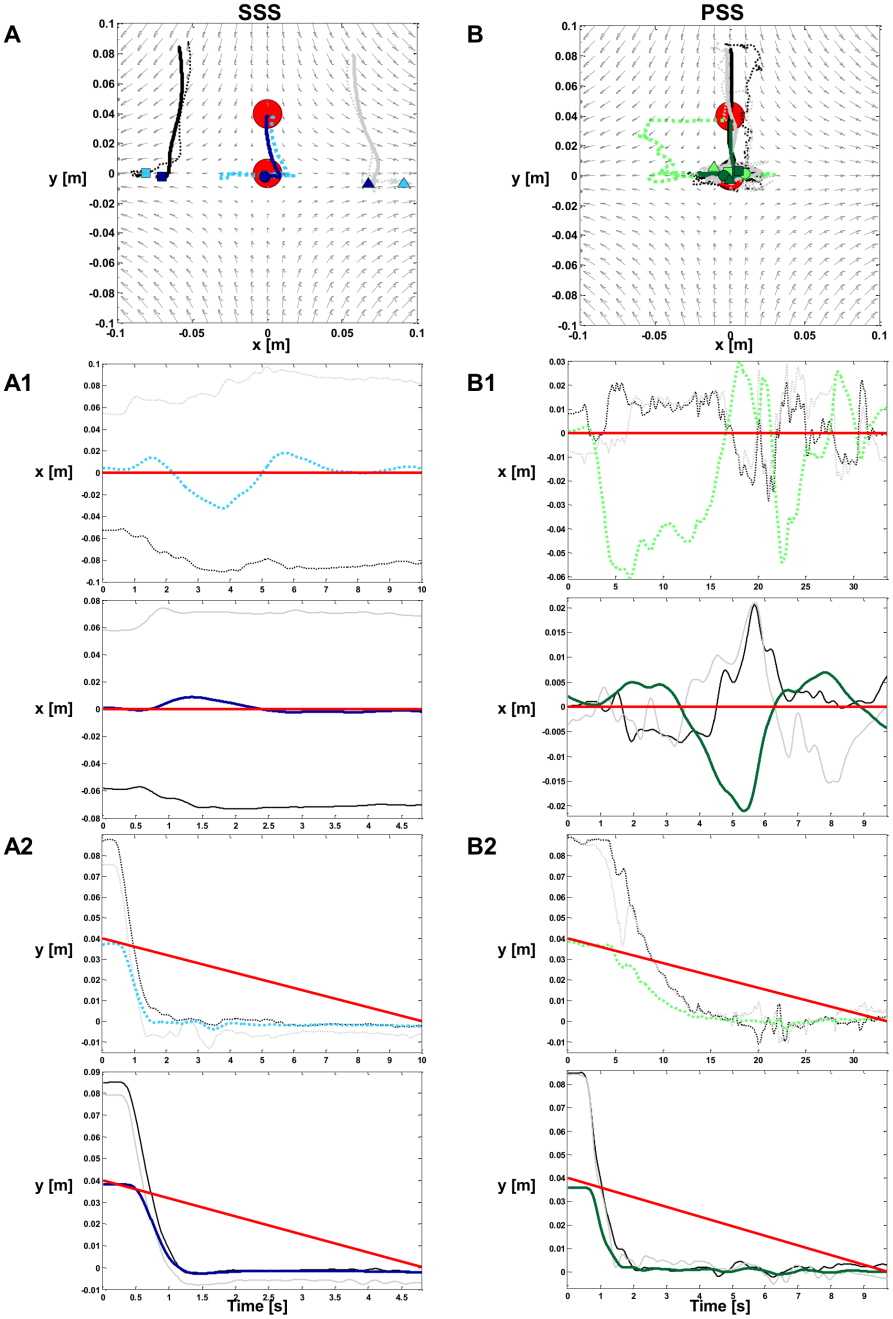
Example of performance in task A (reaching) by subject S1 in the early and final phase of training. Panels A, A1, A2 refer to the SSS and panels B, B1, B2 to the PSS. In particular for the SSS: hand trajectories (black and gray lines), virtual mass trajectory (blue lines), final positions of the left hand (blue square), right hand (blue triangle) and virtual mass (blue circle). For the PSS: hand trajectories (black and gray lines), virtual mass trajectory (green lines), final positions of the left hand (green square), right hand (green triangle) and virtual mass (green circle). In both cases the tool-tip is initially at equilibrium in the forward target and the task is to move it to the central target. The panels show the evolution of the tool-tip and the two hands in different manners: in the workspace (panels A and B) and as functions of time (panels A1, B1 for the *x*-component and A2, B2 for the *y*-component). The curves related to the early phase are dotted and the curves related to the final phase of training are continuous. The red lines in panels A1, B1, A2, B2 represent the ideal evolutions of the tool-tip.

The eight subjects succeeded in becoming expert users of the virtual tool in both stabilization strategies. Figure 6 shows a typical example in which subject S1 attempts to shift the equilibrium of the end-point of the tool from the forward target back to the central target. Panels A, A1, A2 refer to the SSS and panels B, B1, B2 to the PSS; dotted curves are related to the early phase of training and continuous curves to the late phase. In the initial state the two hands overlap and are either positioned just above the initial target (PSS) or are separated sideways and upward in a mirror symmetric manner with respect to the *y*-axis (SSS). In the final state, after reaching the central target, the two hands overlap in the center (PSS) or are aligned symmetrically along the *x*-axis (SSS). The transient state from the initial to the final configuration is characterized by a loss of equilibrium in the initial part of the movement and by a recovery of a new equilibrium in the final part. A slight imbalance between the two hands during the transient may induce large oscillations, particularly if the PSS is used. However, it is apparent that such oscillations, quite visible in early training, tend to disappear when the user masters the tool.

In particular, panels A, A1, A2 illustrate the performance of the subject when he/she operates according to the SSS. Panel A displays the trajectories in the *x-y* plane; panel A1 shows the time course of the *x*-coordinates of the two hands and the tool tip; panel A2 shows the corresponding time course of the *y*-coordinates. The three panels report the motion patterns recorded both in the first target-set, after the familiarization phase, and in the final target-set, when the subject could be qualified as an “expert user”. At the beginning of the coordinated movement pattern, the two hands are beyond the target and well-separated sideways. Thereafter, during the programmed movement of the end-point of the virtual tool, the subject keeps the sideway separation of the two hands while shifting the tool-tip towards the stable manifold of the field. In the first target set, the small coordination error causes a rightward deviation of the virtual mass that then misses the target area. The error is diminished by further increasing the lateral separation of the hands, which in turn increases both stiffness and effort. Ultimately, in the last target-set, the control pattern is similar but better calibrated and thus the equilibrium in the central target is achieved in the first attempt, without a further increase of stiffness.

Panels B, B1, B2 illustrate the performance of the same subject when asked to operate according to the PSS. The initial configuration is quite different with respect to the previous case: the two hands are overlapped and well beyond the target, in such a way that both elastic linkages are aligned with the local field. During the intended movement, the lateral separation of the two hands remains rather small but the inaccuracy of the motion in the first target-set determines an imbalance that pushes the virtual mass on the left, without a significant compensatory movement of the two hands, at least in the first part. The lateral error is corrected by means of a number of correction bursts that finally succeeds to constrain the postural oscillations within the target, as required by the task. In the final target-set, the coordination pattern is similar but clearly more accurate because the target is hit in the first movement and few lateral adjustments, with the paired hands, are necessary to stabilize the posture.

**Figure 7:**
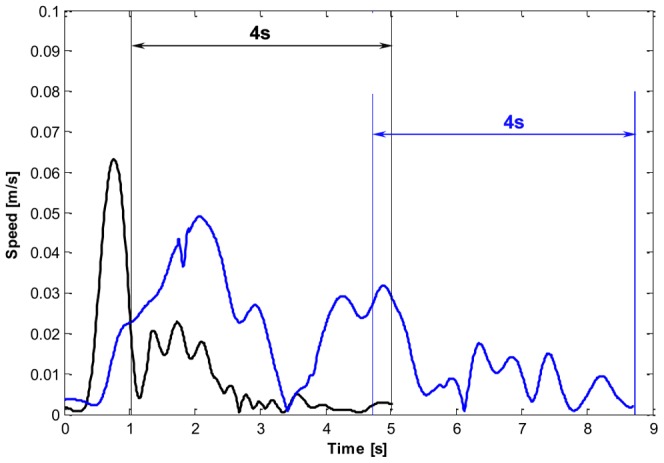
Task A: typical speed profiles of the tool-tip. The figure shows typical speed profiles of a well-trained subject when using the two strategies (SSS: black curve; PSS: blue curve). The initial time corresponds to the presentation of a new target for both strategies. Both profiles include the time-to-target (about 1 s for SSS and 4.8 s for PSS) and the 4 s dwell time, during which residual oscillations of the tool tip remain inside the target area.

Figure 7 shows a complementary aspect of the expert user performance that concerns the typical speed profiles of the tool-tip. In particular, we can observe that after the subject hits the target and succeeds to keep the tool-tip inside the target area for the prescribed duration, the residual oscillations tend to be damped out in the SSS (asymptotic stability), whereas they persist in the PSS, highlighting a bounded stability regime.

**Figure 8:**
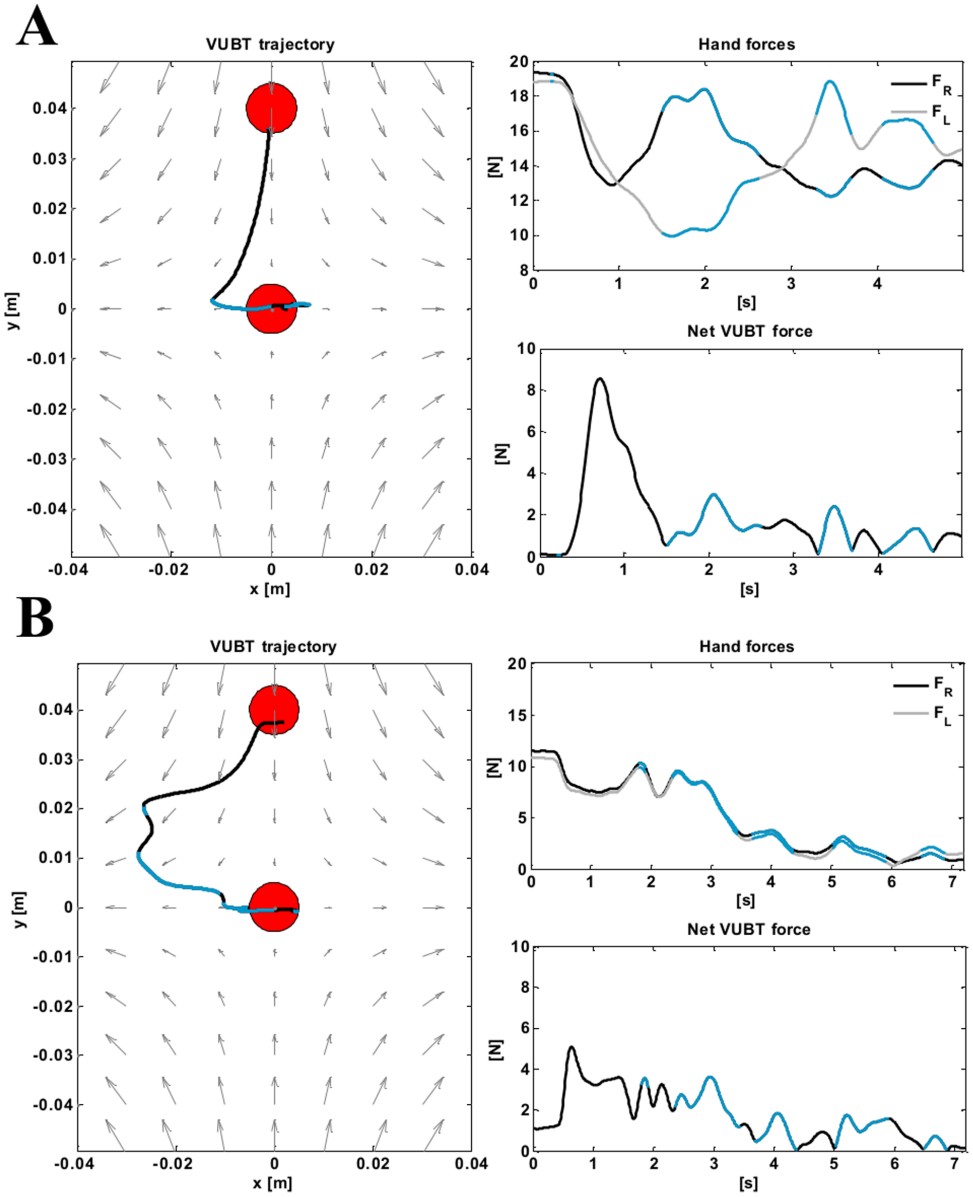
Task A: typical trajectory and force profiles during corrective motions. The figure shows a transient motion between two stabilization phases for S4 in the case of the SSS (panel A) and the PSS (panel B). Left graphs represent the tool-tip trajectory (in black); red circles represent the initial (top circle) and final (bottom circle) target areas; gray arrows represent the saddle-like force-field. The top-right graphs represent the forces imposed by the hands: the right hand is in black, the left hand in gray. The bottom-right graphs depict the resultant of the forces acting on the virtual mass. In every figure, the light blue portions of the signals correspond to corrective motions that contrast the local field, so that the projection of the total force along the direction of the force-field has opposite direction with respect to 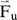.

Besides the kinematic performance, it is relevant to observe how the user copes with the loss of equilibrium during the transient from a dynamic point of view. In particular, we expect that the undertaken corrective actions will differ according to the adopted motor strategy. Figure 8 shows a representative example, in which S4 has to bring and stabilize the virtual tool from the topmost target into the origin (red circles) of the saddle-like force-field, first following the high stiffness strategy (panel A) and later the positional strategy (panel B). As previously explained, when the equilibrium conditions are not met, the resultant of the forces acting on the virtual tool is not null, causing the mass to move (black trajectory in the left graphs of Figure 8). In this case, the subject can either control the motion of the ball by applying two forces 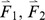 that interfere with the local field 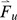 or let the virtual mass move according to 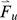. In particular, in Figure 8, the portions of the signals corresponding to corrective motions that contrast the local field (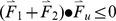) are highlighted in light blue. The resultant of the forces is reported in the graphs entitled ‘Net VUBT force’. As it can be noted, under both strategies, the net force is maximal at the beginning of the trial and tends to decrease to zero during the stabilization phase (last 4 s). The difference between the two conditions in panel A (SSS) and B (PSS) resides in the magnitude of the forces applied by the hands. In the case of the SSS, the hands tend to apply symmetrical forces with respect to the y-axis up to the point in which the local field makes the virtual mass deviate from the vertical line connecting the two targets. At this point, the hand forces an imbalance in favor of the right hand to contrast the leftward shift in position imposed by 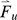 and bring the tool inside the target area. Successively the left hand acts to damp the oscillations of the tool around the equilibrium position. In the case of the PSS, the two hands always act synchronously, as we expected. Despite the system being less rigid than in the SSS, the positional strategy is effective in controlling the tool-tip position and requires the subject to apply forces of much lower intensity.

**Figure 9:**
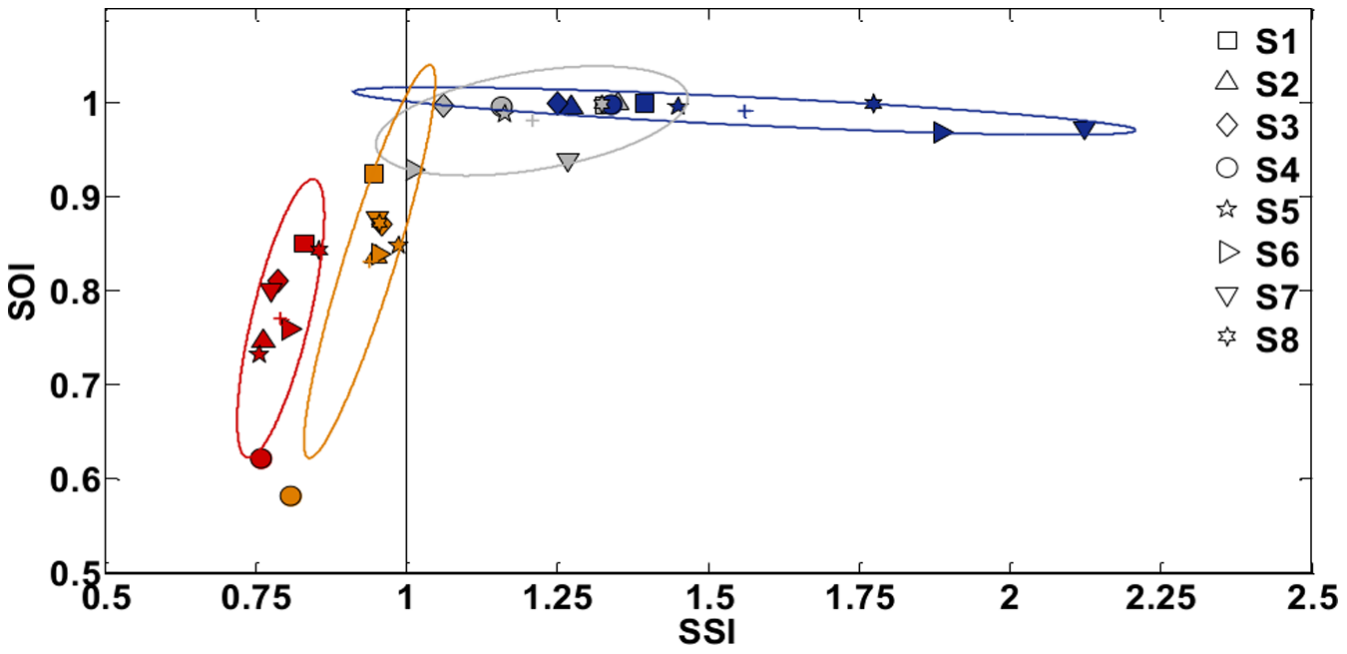
Task A: *SOI* (Stiffness Orientation Index) vs. *SSI* (Stiffness Size Index). Effect of training for becoming expert users. The displayed average values are related to the early phase of training (SSS: blue; PSS: gray) and the final phase (SSS: orange; PSS: red), for all the eight subjects (S1 –S8). The shaded area represents the ellipse of variation along the principal components over the four conditions; ‘+’ symbols are the centroids of the distributions.

**Figure 10:**
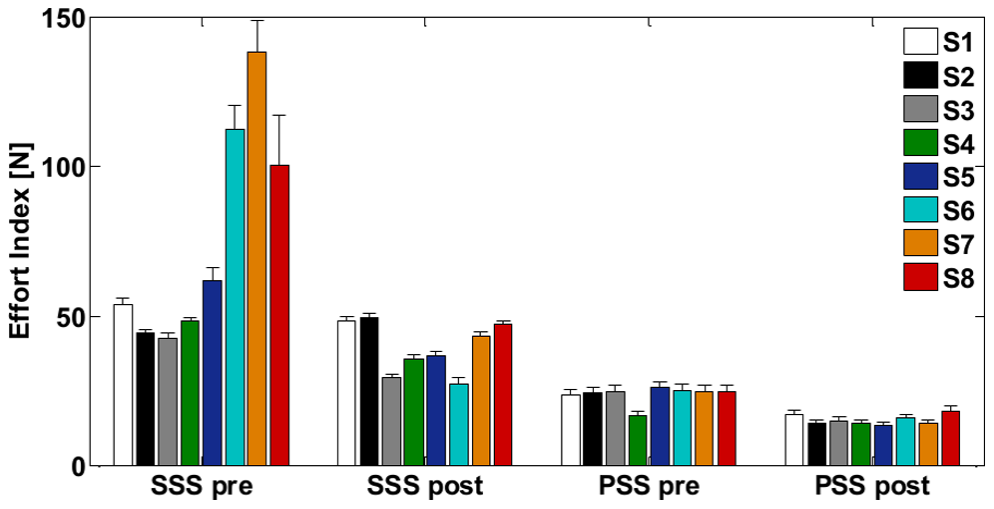
Task A: *Effort Index (E)*. Mean effort employed by the subjects (S1 – S8) for the two strategies (SSS and PSS) during the early phase of training (SSS pre, PSS pre) and the final phase (SSS post, PSS post); whiskers represent the corresponding standard errors.

**Figure 11:**
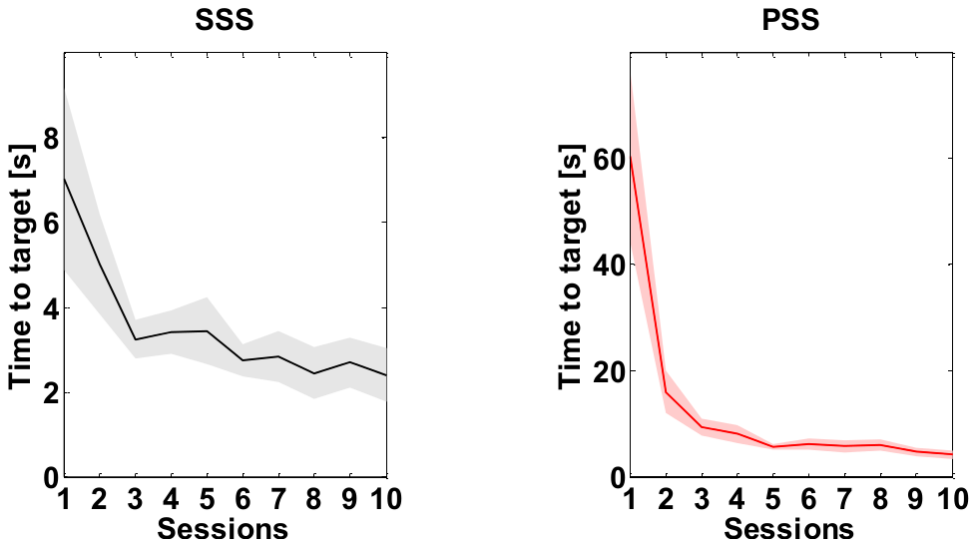
Task A: *Time to Target (TT)*. Evolution of the *TT* index throughout the ten sessions of training. The continuous line is the mean value over the eight subjects while the shaded area corresponds to the standard error. Left panel refers to the SSS while right panel to the PSS one.

**Figure 12:**
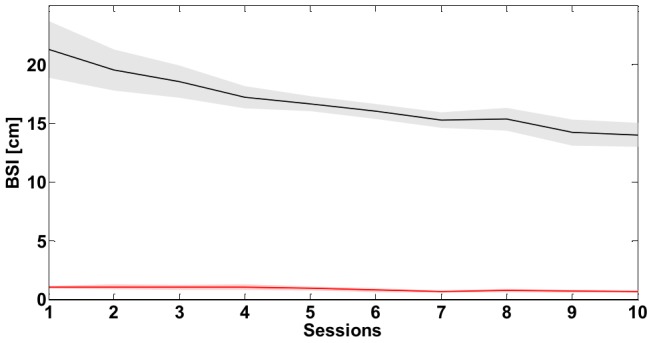
Task A: *Bimanual Separation Index (BSI)*. Evolution of the *BSI* throughout the ten sessions of training. The continuous line is the mean value over the eight subjects while the shaded area corresponds to the standard error. The black line refers to the SSS while red one to the PSS.

**Figure 13:**
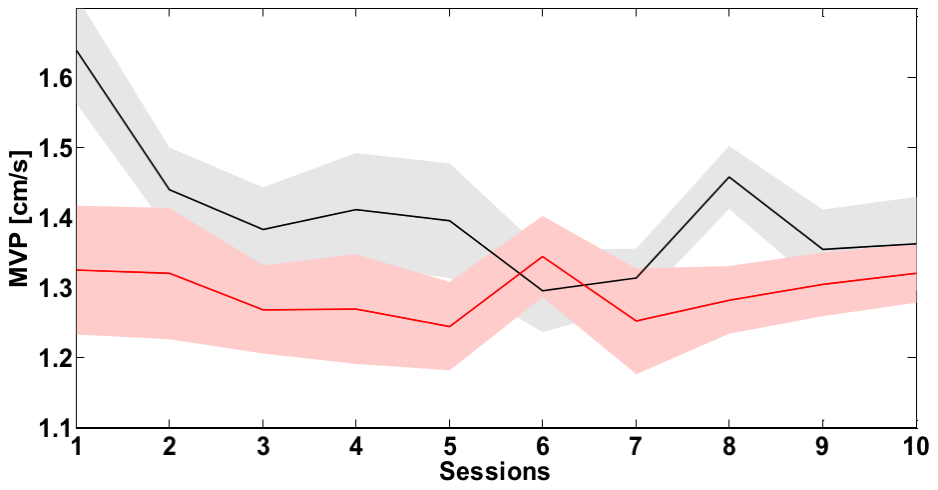
Task A: *Magnitude of the Velocity Peaks (MVP)*. Evolution of the *MVP* of the virtual mass throughout the ten sessions of training. The continuous line is the mean value over the eight subjects while the shaded area corresponds to the standard error. The black line refers to the SSS while red one to the PSS.

Figures 9-13 show the effect of training in the different indicators defined in the methods for the two strategies and the eight masters. Let us first consider the five indicators that assess the performance during the reaching phase of the task (*SSI, SOI, E, TT, BSI*). We will describe the indicators referring to the stabilization phase (*MVP, FVP)* thereafter.

Figure 9 shows the relation between the Stiffness Size index and the Stiffness Orientation Index when the subjects are asked to adhere to one strategy or the other, in the first compared to the last session of the training. The vertical line (*SSI* = 1) corresponds to the situation in which the bimanual coordination is such that the medio-lateral component of the stiffness matrix of the virtual tool balances the instability coefficient of the force-field. On the left of that line, the stabilization strategy is classified as PSS; on the right it is classified as SSS. Already immediately after the familiarization phase, all the subjects succeed in satisfying the task requirements, distributing on the right hemiplane when performing the SSS (blue ellipse – centroid  =  [1.56; 0.99]) and on the left one during the PSS (orange ellipse – centroid  =  [0.94; 0.83]). Moreover, we observe that at the end of training all the subjects reduced their operating stiffness and consequently the *SSI* as well as the employed effort (SSS - gray ellipse: centroid  =  [1.21;0.98], PSS - red ellipse: centroid  =  [0.79;0.77]). If SSI decreases also the degree of stretching of the two springs decreases, thus reducing the required effort. Globally, we notice that the variability among subjects related to the *SSI* is much reduced at the end of the training (SSS pre = 0.13; SSS post = 0.05; PSS pre = 0.03; PSS post = 0.01) indicating the accomplishment of similar performance. To analyze the results in Figure 9 more in-depth, we compared the Stiffness Size Index and the Stiffness Orientation Index values among the two strategies (SSS, PSS) both in the beginning (pre) and in the end (post) of the training. In particular, according to the Mann Whitney *U* test, SSI values in the two strategies differ significantly, independent of the skillfulness level (SSS pre - PSS pre and SSS post - PSS post: *U* = 0, *n* = 8, *p*<0.001 two-tailed): median [IQR] values are 1.43 [0.52] for SSS pre and 0.94 [<0.01] for PSS pre (first comparison), 1.21 [0.22] for SSS post and 0.79 [0.06] for PSS post (second comparison). The same applies to the SOI values: median [IQR] values are 0.99 [0.01] for SSS pre and 0.83 [0.04] for PSS pre (first comparison), 0.98 [0.03] for SSS post and 0.77 [0.03] for PSS post (last comparison). In addition, the training determined a significant variation in the SSI indicator medians according to the Wilcoxon matched pairs signed-rank test in both conditions (SSS pre-post: *p* = 0.025; PSS pre-post: *p* = 0.012) and in the SOI indicator medians in the case of PSS strategy (SSS pre-post: *p* = 0.069; PSS pre-post: *p* = 0.025).As already suggested by Figure 9, Figure 10 highlights a consistent reduction in the effort employed by the subjects in both strategies as a consequence of the training (SSS: −47.2%, *p* = 0.017, *T* = 1; PSS: −34.0%; *p* = 0.012, *T* = 0), resulting from a Wilcoxon match pairs signed-rank on the effort values in each strategy comparing the first and the last sessions. However, in the SSS case the level of effort (median [IQR]: 40.17±15.07 N) remains significantly higher (Mann Whitney *U* test: *U* = 0, *n* = 8, *p*<0.001 two-tailed) than in the case of PSS (median [IQR]: 14.50±2.53 N).

One may expect a trade-off between the effort and the time required to reach the target when switching from one strategy to the other and this is clearly the case. Figure 11 shows the evolution of the Time to Target indicator over the training. In both conditions the indicator reaches a steady state value that, despite being inferior for the SSS (median [IQR]: 1.97 [0.44] s) with respect to the PSS (median [IQR]: 3.77 [4.86] s), is not significantly different between the two strategies (Mann Whitney U test: *U* = 13, *p* = 0.052 two-tailed). The fact that reaching time under the SSS, condition is lower can be explained considering that in the SSS the environmental instability is ‘hidden’ by the high stiffness value of the tool, although at the expense of great effort. In contrast, the reaching movements of the PSS case, e.g. the movement illustrated in Figure 6, require concurrent active control along the *y*-axis for approaching the target and active generation of stabilization bursts along the *x*-axis for avoiding instability, lengthening the time required for achieving both goals.

The analysis of the Bimanual Separation Index (Figure 12) complements the overall picture illustrated by the analysis of the other indicators. As expected, the figure shows that the *BSI* is markedly different in the two strategies and is much higher in the SSS case (always above 13 cm of difference). In particular, we observe that the training reduces the separation between the two hands in the stiffness strategy only (−7 cm), partially explaining the reduction of effort in Figure 10. The *BSI* difference across conditions is relevant to ascertain that all the subjects executed the task correctly and they didn't overlap the two strategies during the training. Indeed, in the SSS case they learned to keep the two hands sufficiently separated sideways, whereas in the PSS case they understood that the two hands should be considered as a single unit and focused on fine, synergic stabilizing corrections.

As a next step, it is worth characterizing in a quantitative manner the “postural component” of the task, namely the process by which the subjects succeed in constraining the “sway movements” of the end-point of the virtual tool (i.e. the virtual mass) inside the target area, during the stabilization interval. This process must be active in both strategies: this is obvious both in the PSS case, because the virtual tool is operated in a condition of mechanical instability, and in the SSS case, because the intrinsic muscle noise associated with the high effort level can generate fluctuations that need to be compensated, despite the mechanical stability of the virtual tool. To better highlight differences in the two control strategies, we analyzed the Magnitude of the Velocity Peaks and Frequency of the Velocity Peaks indicators both for the virtual mass speed and the hands speed.

Figure 13 displays the time course of the *MVP* indicator computed on the virtual mass speed profile throughout the training. At the beginning, the SSS required the subject to perform grosser corrections to keep the tool-tip inside the target position (M ± SE: 1.64±0.08 cm/s), if compared to PSS (M ± SE: 1.33±0.09 cm/s). This difference holds until learning advances: after the 6^th^ –7^th^ session, such a difference tends to disappear (M ± SE session 10: SSS 1.36±0.07 cm/s; PSS 1.32±0.04 cm/s). Noticeably, the same sessions correspond to the initial plateau phase for the time to target indicator in the SSS case, denoting a consistent learning. This kind of invariance at the final stages of training can be partly explained by the design of the task, namely the size of the target in relation to the intensity of the force-field. On average, the *MVP* data computed in the case of the mass are not significantly different between the two strategies (one-way ANOVA with strategy as factor, subjects as random effect, *p* = 0.068). Interestingly, the employed stabilization strategy significantly influences the *MVP* computed on hands speed (one-way ANOVA with strategy as factor, hand as nested factor, subjects as random effect, *p*<<0.001, *F* = 37.71, *partial η^2^* = 0.64). In particular, the *MVP* value is not dependent upon the considered hand (*p* = 0.56, *F* = 0.60) and tends to be much greater in the PSS (M ± SE  =  2.28±0.11 cm/s) than the SSS (M ± SE  =  1.75±0.07 cm/s). This should not be surprising, since it strongly depends on the stiffness properties of the VUBT. The tool in the postural condition is characterized by a much lower stiffness that slows down the response of the system to perturbation applied at the hands. Hence, we may expect that the frequency of the corrective actions performed by the hands in the PSS is lower than that in the SSS. Indeed, when considering the Frequency of the Velocity Peaks indicator the two strategies strongly differ (*p*<<0.001, *F* = 199.60, *partial η^2^* = 0.90), where the mean *FVP* (M ± SE) is equal to 2.17±0.02 Hz in the PSS and 2.37±0.01 Hz in the SSS, and is not dependent on one particular hand (*p* = 0.23, *F* = 1.58). This difference is amplified when considering the mean oscillations of the virtual mass velocity that are remarkably slower in the PSS (*p*<<0.001, *F* = 34.47, *partial η^2^* = 0.71; M ± SE: PSS: 1.24±0.02 Hz; SSS: 1.43±0.02 Hz). Therefore, the results obtained for the *MVP* and the *FVP* indicator support the idea that similar postural performance levels can be achieved by strongly different control strategies.

**Figure 14:**
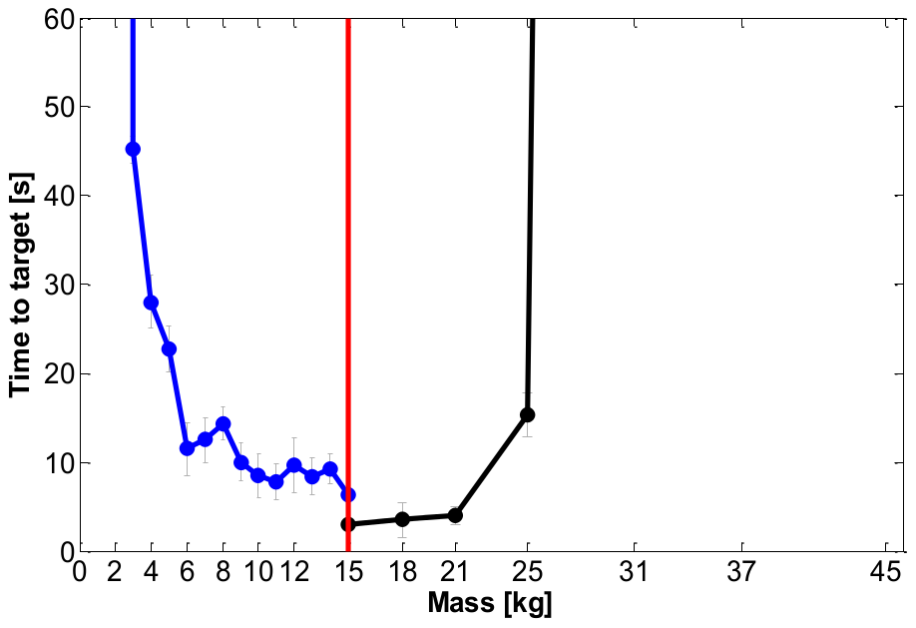
Task A: sensitivity of a performance parameter (*TT, Time to Target*) to variations of the virtual mass. The blue curve corresponds to the PSS: in this case, the damping factor ζ is maintained constant and the values of the virtual mass and of viscosity are reduced systematically from the nominal values, with a dramatic reduction of performance when the mass is reduced below 6 kg. The black curve corresponds to the SSS: in this case the damping factor ζ is reduced in steps from 0.7 to 0.1, by increasing accordingly the value of the virtual mass, with a dramatic breakdown of performance when the mass exceeds 25 kg. The data represent mean values (with the corresponding standard errors) for the subject that performed the sensitivity task.

Finally, let us consider the result of the sensitivity analysis carried out on a subject at the end of his training, according to the criteria described in the methods. For the PSS we considered systematic reductions of the virtual mass from its nominal value of 15 kg, while maintaining the damping factor of the virtual tool fixed. For the SSS we systematically increased the virtual mass while reducing the damping factor. Among the different performance indicators we focused on *TT* because it clearly identifies the difficulty in achieving the task. Figure 14 plots the variations of *TT* when *M* is increased (SSS) or decreased (PSS), indicating that a trained user of the virtual tool can manage a variation of the virtual mass in a rather large range, namely between 4 kg and 25 kg. This result can be attributed to the generalization capability of the trained subject. Moreover, it confirms the rationale of the design of the virtual tool, described in the method, in the sense that the choice of the tool parameters appears to be rather “neutral”, with regard to the choice of stabilization strategy, and close to optimal, with regard to the time-to-target.

### Task B

The objective of this task was to test, in a preliminary way, the degree of generalization that could be achieved by the expert users when faced with a more complex exercise: tracking smoothly moving targets, along a variety of paths, instead of reaching a small set of targets. The intrinsic dynamics of the environment implies coordination and stabilization patterns that change continuously from point to point of the workspace. Thus, it is unlikely that the extensive training in the reaching task allowed the subjects to accumulate the “dense” experience of the environmental dynamics that is required in the task B.

The metrics used for evaluating the generalization capability is based on the tracking performance and how the subjects comply with the task by using the learned strategies.

The subjects were not encouraged to prefer one strategy or the other during the overall tracking or parts of it, but a working hypothesis is to expect all of them to choose the stiffness strategy as the preferred one when moving outside the peanut-shaped marginal stability region. On the contrary, the adopted strategy might vary among subjects when the tip of the virtual tool is moving inside the boundary of that particular region.

In order to elucidate the actual performance, we first computed the *SSI* to discriminate between the two strategies: *SSI* < 1  =  *PSS*, *SSI* > 1  =  *SSS*, with a tolerance of 10% around the boundary. Secondly, we sought for any particular distribution of the strategies along the trajectory in each of the subtasks to assess if the choice of a strategy could be accounted for by the properties of the workspace region in which the end-tool was actually moving. Finally, we compared this indicator with the tracking error and its time derivative so as to investigate if the chosen strategy could account for differences in the tracking performance.

The analysis of the tracking error indicator in Table 3 shows that, in the case of subtask B1 and B2, the subjects tend, on average, to improve their tracking abilities in time. Considering the final lap, the mean tracking error along the whole trajectory is always inferior to 2.15 cm, suggesting that skilled subjects succeeded in tracking the moving target within the prescribed time in these two simpler subtasks. The same is not true when considering untrained subjects, who could not fulfill the exercise requirements in any of the four subtasks. Considering subtasks B3 and B4, the performance of the subjects vary from lap to lap. For instance, the tracking error for subject S8 in B4 varied from 1.01 cm (lap 1) to 4.18 cm (lap 3). This can be imputed to the choice of the strategy to employ inside the region of marginal stability and to the greater variability in the local unstable field direction along the trajectory.

**Table 3:** Performance Indicator in Task B.

	Tracking Error [cm]				
Subjects	Lap	B1	B2	B3	B4
S1	1	2.73±1.87	1.43±1.07	1.99±1.42	1.28±0.82
	2	1.96±2.19	0.90±0.55	1.53±1.20	1.70±1.36
	3	1.36±1.01	1.38±1.00	1.79±1.40	1.50±1.12
S4	1	2.84±1.49	0.94±0.81	1.63±1.21	1.53±0.82
	2	1.81±0.96	1.33±0.86	1.81±1.19	1.72±1.12
	3	1.26±0.67	0.92±0.74	1.96±1.43	2.16±1.31
S5	1	2.44±1.27	2.12±1.50	1.02±0.62	2.72±1.57
	2	1.71±0.90	2.36±1.22	1.10±0.90	0.92±0.51
	3	1.55±0.73	1.97±1.58	1.25±0.79	1.13±0.60
S6	1	1.91±0.89	1.83±1.15	2.49±2.30	3.68±2.87
	2	3.57±3.04	1.31±0.94	2.28±1.94	4.85±3.36
	3	2.07±1.09	1.58±0.88	4.08±3.33	1.77±0.96
S7	1	1.24±0.78	0.69±0.38	0.89±0.52	0.68±0.30
	2	1.40±0.89	0.51±0.37	0.86±0.50	1.62±1.20
	3	1.45±0.97	0.64±0.51	0.90±0.55	1.21±0.79
S8	1	2.52±1.44	3.51±2.70	3.68±2.85	1.01±0.54
	2	2.45±1.19	4.52±2.70	3.46±3.46	3.92±2.47
	3	2.14±1.71	0.99±1.23	2.92±2.55	4.18±2.63

**Figure 15:**
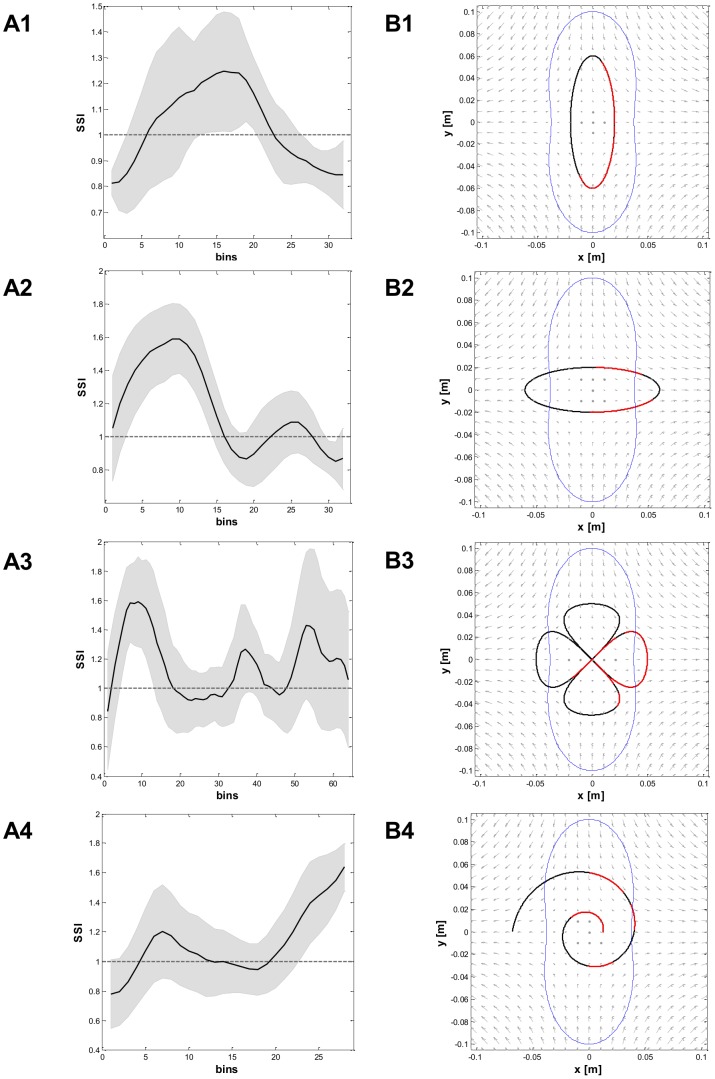
SSI evolution and desired trajectories for task B. **Panels A1,A2,A3,A4:** mean SSI evolution over time (in black) for the four cases in task B in the case of PSS >20%. In gray is represented the standard deviation. **Panels B1, B2, B3, B4:** desired trajectories for the four cases in task B. The target trajectory is highlighted in red, whenever PSS was the preferred strategy on average while in black in the SSS case. In all the panels in the background there is the force-field and the locus of marginal stability (in blue).

**Figure 16:**
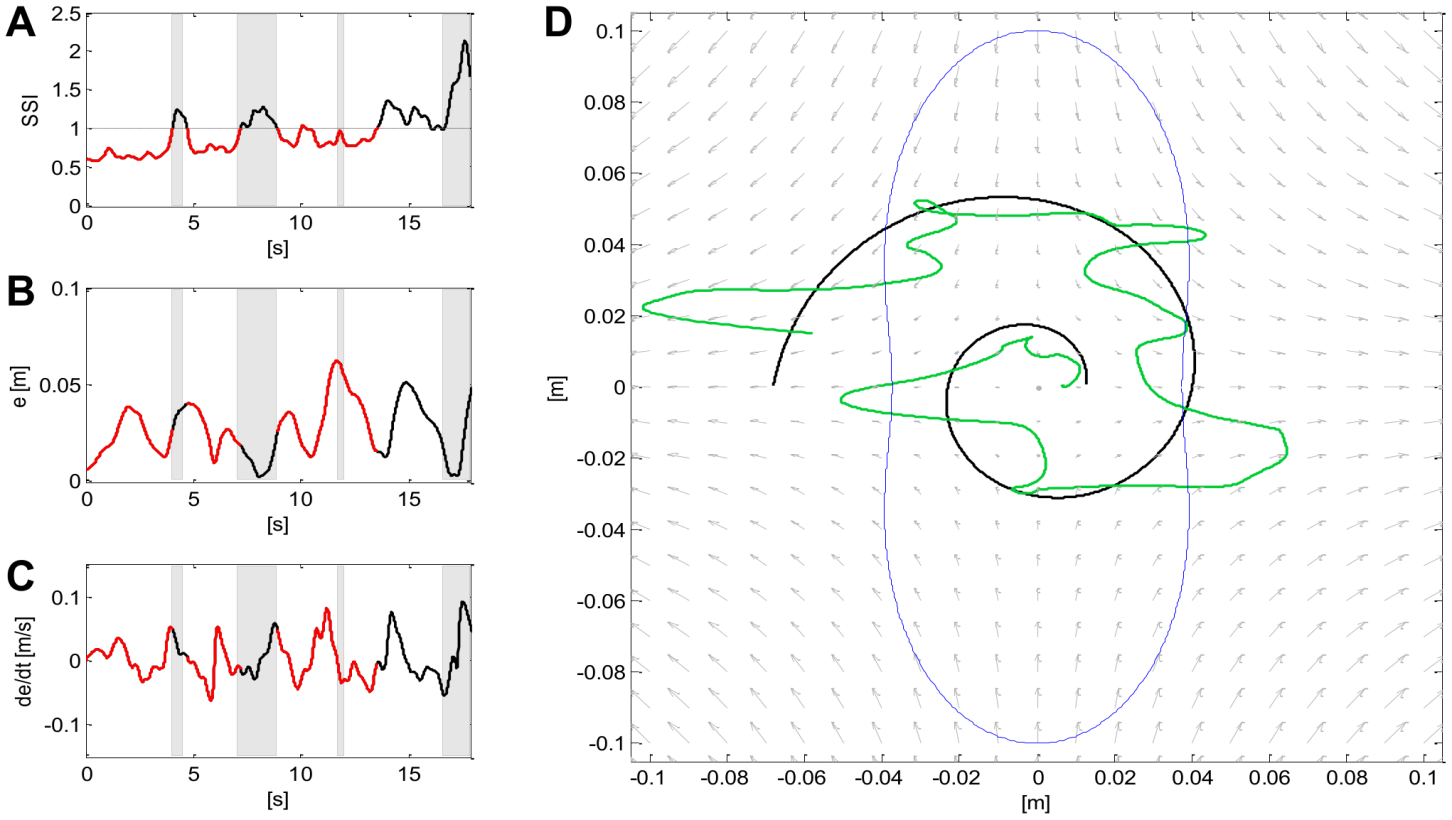
Overall performance of subject 4 for subtask B4. It summarizes the performance of S4 for lap 1 of subtask B4. **Panel A:** Evolution over time of the SSI. **Panel B:** Evolution over time of the tracking error. **Panel C:** Evolution of the time derivative of the tracking error. **Panel D:** Tool-tip (in green) and desired trajectory (in black). In panels A, B, C, the red portion shows the use of the PSS while the black one the use of the SSS. The gray shaded areas represent time intervals during which the tool-tip exceeded the marginal stability region (blue line).

To assess which strategy was active in the different phases of the tracking, we computed the *SSI* and the percentage of PSS choice throughout each trial, reported in Table 4. By taking a closer look at these data, we can observe that S7 never adopted the PSS, while all the other subjects showed no preferred strategy when the target was moving inside the marginal stability region and demonstrated capability to freely switch from one strategy to the other. However, when the target was sliding away from that region, the strategy choice was forced towards the SSS, as our hypothesis suggested. This behavior is highlighted in Figure 15, which summarizes the mean *SSI* evolution over time (panels A1, A2, A3, A4) and the desired trajectory (panels B1, B2, B3, B4) for the four subtasks in the case of PSS>20%. The target trajectory is highlighted in red, whenever PSS was the preferred strategy on average. Subjects demonstrated similar strategic patterns throughout each tracking subtask. However, the *SSI* standard deviation tends to increase in correspondence to the portions of path for which both strategies can be regarded as equivalent from a stabilization point of view. Figure 16 addresses this issue more in detail by focusing on the performance of S4 (panels B, C, D) and strategy choices (panel A, red  =  PSS, black  =  SSS) when executing subtask B4 for the first time (Lap 1); the executed trajectory is depicted in green in the panel D of the same figure. The gray shaded areas represent time intervals during which the tool-tip exceeded the marginal stability region (blue line in panel A). As can be noticed, S4 applied both the PSS and SSS, but the latter was preferred every time the bounded stability conditions were not met (i.e. over the shaded areas). Not only was the subject able to switch to SSS strategy whenever imposed by the task dynamics, but the chosen strategy was also modified independently from the forced stability requirements (*SSI* in the 12 to 17 s interval).

**Table 4:** Strategy switching in Task B.

	Percentage of SSI <1											
Subjects	B1			B2			B3			B4		
	Lap 1	Lap 2	Lap 3	Lap 1	Lap 2	Lap 3	Lap 1	Lap 2	Lap 3	Lap 1	Lap 2	Lap 3
S1	60.62	21.54	42.78	20.19	19.94	17.64	45.31	25.07	38.74	19.79	27.32	35.00
S4	39.68	29.89	37.33	28.14	39.03	25.99	57.04	52.19	57.26	62.58	58.54	52.12
S5	17.79	13.29	0	28.14	26.49	37.43	7.80	13.25	24.94	37.28	3.56	4.51
S6	3.00	9.50	9.00	39.18	42.08	42.68	4.22	14.87	28.49	10.64	43.24	15.69
S7	0	0	0	0	0	0	0	0	0	0	0	0
S8	0	1.25	13.44	22.04	13.59	0.50	21.24	9.17	29.14	0	50.75	34.22

As a point of interest, we report that switching from PSS to SSS was associated to a reduction in the rate of growth of the tracking error in the great majority of the cases (e.g. *SSI* for t = 4 s). This consideration suggests that our brain might adopt the stiffness strategy as a way to reduce errors due to inefficacy of intermittent control. Indeed, when dealing with uncertainties in the environment, increasing the end-point stiffness improves stability and provides a better control over the position in time [22].

## Discussion

The experiments have demonstrated that if a subject is required to stabilize an unstable load and the dynamics of the load allows for two different control strategies, namely a high-stiffness/high-effort strategy and a low-stiffness/low-effort strategy, suitable training is sufficient to master both strategies and to switch from one to the other. In general, this is a preliminary but relevant piece of information to understand how the brain can switch among different strategies during complex motor tasks, such as when car drivers switch from one gear to another in different driving conditions. At the same time, the knowledge of such mechanisms is important from the point of view of ‘human factors’ in the design of ergonomic man-machine interfaces that involve compliant interaction and instability.

It is also remarkable that, for both strategies, the subjects were able to generalize the knowledge stored in the learned coordination/control models even though the combination of force intensity, force direction, bimanual coordination, and appropriate stiffness in the different parts of the workspace is variable in a continuous manner. Thus training in a discrete task, like reaching a limited number of targets, was good enough to achieve almost immediately good performance also in a continuous task-like tracking.

In most of the studies on force-field adaptation, since the seminal paper by Shadmehr & Mussa Ivaldi [28], the focus is on movement more than postural stability, as related to the mechanisms of initiation and termination of movement. Although this is somewhat understandable when dealing with adaptation to stable dynamics, it is less so with unstable dynamics [9], [10], [29]: posture and movement, in general, are related but are characterized by highly specific features as it is evident, for example, in the antinomy between gait and standing posture. In the case of arm movements, the posture/movement distinction is particularly strong if we consider discrete, fast movements whereas it is somehow blurred in the case of continuous, relatively slow tracking movements. In any case, we believe that isolating posture from movement, for example by switching on/off the unstable force-field at the beginning/termination of movements, as in [9], is likely to break down the ecological continuity of purposive actions and introduce an element of artificiality in the experimental paradigm. For this reason we think that in the study of adaptation to unstable environments the subjects should be exposed persistently to a source of instability, as we have done in our experiments.

Along a similar line of thinking, we believe that questions about interference in a learning independent internal model, for adaptation to different dynamics and visuomotor transformations [30], [31], [32], [33], [34], are somewhat artificial because what really matters, from the ecological point of view, are purposive actions, not a set of movements or internal models. From this point of view, we agree with the emphasis on the importance of context, for integrating in a single control mechanism multiple model, which has been advocated, for example, by Howard et al. [35].

The type of control applied by the expert user to the virtual compliant tool is basically positional bimanual control. Only in this way would the stiffness properties and the intrinsic dynamics of the tool, while interacting with the unstable environment, allow for predictions to be made. In fact, whatever the chosen stabilization strategy, the subject must figure out how to stretch the two non-linear springs, namely where to put the two spring terminals, in order to compensate intensity and direction of the local field at the current target position. This is clearly a feedforward computation, reflecting a model of the force-field acting on the tool. However, compensating the disturbing effect of the field (in a feedforward manner) is only one side of the required answer: the other side is stabilization, either asymptotic or bounded. In the former case, we are again in the presence of a feedfoward mechanism, which must memorize the degree of lateral stretching of the two spring terminals which is appropriate in different parts of the workspace. In the latter case, when the two spring terminals are kept quite close to each other (with a very small value of the Bimanual Separation Index) the required control paradigm is feedback positional control, quite similar to the stabilization of a manually-held inverted pendulum or the human upright posture: in both cases, the recorded postural sway patterns can be attributed to intermittent feedback control [23], [24], [25], [41]. Thus feedforward, feedback, and stiffness control paradigms are deeply interrelated in the investigated experimental environment, in a similar way to real life tasks that entail tools interacting with material properties of manipulated objects. The “hybrid” nature of such interaction paradigms is not compatible with a global optimization scenario, but rather suggests a local scenario, in agreement with the work of Ganesh et al. [42], who addressed the issue of sub-optimality in motor planning and the role of “motor memory” in consolidating the choice of a suboptimal strategy, by showing the role of motor memory in the local minimization of task-specific variables. In our case, for example, when the subjects switch to the SSS they also implicitly make a sub-optimal choice, because they orient the stiffness ellipse in the optimal way, given the strategy, namely with the long axis aligned to the unstable manifold of the field. In contrast, when they switch back to the PSS they also abandon the stiffness orientation issue and focus instead on appropriately timed correction bursts that can provide bounded stabilization. In regard to the issue of a possible “meta-strategy” of strategy switching in task B, we may say that the data suggest a tendency to prefer SSS when the target exits the marginal stability area (see Figure 16), whereas there are alternations between the two regimes when the target hovers inside that region, without any clear selection criterion. However, in the latter case, it appears that, from the data, the switching mechanism is rather slow, in the sense that when a strategy is selected it remains the same for several seconds. We may speculate that in this case the subjects may play a trade-off between precision and effort. Let us use an automotive metaphor, namely let us compare strategy shifting to manual gear shift in a car, with SSS as a lower and PSS as a higher gear: one may shift down the gear to SSS when he wishes to stay well on target, for example when overtaking another car, and then shifts up to PSS when that requirement is relaxed and/or wishes to avoid overheating the engine and wasting excessive fuel.

A related issue is the identification of the reference frame that best explains force-field adaptation and generalization. The literature in this area is mostly related to stable dynamics and since the seminal paper by Shadmehr & Mussa Ivaldi [28] has concluded that the adaptation process takes place in joint coordinates and is characterized by spatial generalization [36], [37], [38], [39], [40], although a recent study [43] challenges the posited predominance of a single global coordinate system and suggests instead mixed coordinate representations and local learning. Our experiments agree with this “hybrid” representational paradigm also suggesting pairing it with the previously defined “hybrid” control paradigm. Moreover, our experiments go one step further, emphasizing the crucial role of the “tool space”, in addition to the “joint space” and the “end-effector space”, in the representation, organization, and control of the virtual tool in the investigated unstable task. We also suggest that these findings point to the “multi-referential” nature of motor cognition and motor control, also in the context of a computational approach to the concept of body schema, both for humans or humanoid robots [44], [45], [46].

## Supporting Information

File S1
**Combined file of supporting tables and figures. Table S1. Performance Indicators in Task A. Figure S1. Task A: typical trajectory and force profiles during corrective motions.** The figure show a transient motion between two stabilization phases for the subject 1 (S1) for the first session in the case of the SSS (top panel) and the PSS (bottom panel). Left graphs represent the tool-tip trajectory (in black); red circles represent the initial (top circle) and final (bottom circle) target areas; grey arrows represent the saddle-like force-field. The top-right graphs represent the forces imposed by the hands: the right hand is in black, the left hand in grey. The bottom-right graphs depict the resultant of the forces acting on the virtual mass. In every graph, the light blue portions of the signals correspond to corrective motions that contrasts the local field, so that the projection of the total force along the direction of the force-field has opposite direction with respect to 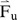. **Figure S2. Task A: typical trajectory and force profiles during corrective motions.** The figure show a transient motion between two stabilization phases for the subject 2 (S2) for the first session in the case of the SSS (top panel) and the PSS (bottom panel). Left graphs represent the tool-tip trajectory (in black); red circles represent the initial (top circle) and final (bottom circle) target areas; grey arrows represent the saddle-like force-field. The top-right graphs represent the forces imposed by the hands: the right hand is in black, the left hand in grey. The bottom-right graphs depict the resultant of the forces acting on the virtual mass. In every graph, the light blue portions of the signals correspond to corrective motions that contrasts the local field, so that the projection of the total force along the direction of the force-field has opposite direction with respect to 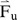. **Figure S3. Task A: typical trajectory and force profiles during corrective motions.** The figure show a transient motion between two stabilization phases for the subject 3 (S3) for the first session in the case of the SSS (top panel) and the PSS (bottom panel). Left graphs represent the tool-tip trajectory (in black); red circles represent the initial (top circle) and final (bottom circle) target areas; grey arrows represent the saddle-like force-field. The top-right graphs represent the forces imposed by the hands: the right hand is in black, the left hand in grey. The bottom-right graphs depict the resultant of the forces acting on the virtual mass. In every graph, the light blue portions of the signals correspond to corrective motions that contrasts the local field, so that the projection of the total force along the direction of the force-field has opposite direction with respect to 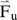. **Figure S4. Task A: typical trajectory and force profiles during corrective motions.** The figure show a transient motion between two stabilization phases for the subject 4 (S4) for the first session in the case of the SSS (top panel) and the PSS (bottom panel). Left graphs represent the tool-tip trajectory (in black); red circles represent the initial (top circle) and final (bottom circle) target areas; grey arrows represent the saddle-like force-field. The top-right graphs represent the forces imposed by the hands: the right hand is in black, the left hand in grey. The bottom-right graphs depict the resultant of the forces acting on the virtual mass. In every graph, the light blue portions of the signals correspond to corrective motions that contrasts the local field, so that the projection of the total force along the direction of the force-field has opposite direction with respect to 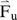. **Figure S5. Task A: typical trajectory and force profiles during corrective motions.** The figure show a transient motion between two stabilization phases for the subject 5 (S5) for the first session in the case of the SSS (top panel) and the PSS (bottom panel). Left graphs represent the tool-tip trajectory (in black); red circles represent the initial (top circle) and final (bottom circle) target areas; grey arrows represent the saddle-like force-field. The top-right graphs represent the forces imposed by the hands: the right hand is in black, the left hand in grey. The bottom-right graphs depict the resultant of the forces acting on the virtual mass. In every graph, the light blue portions of the signals correspond to corrective motions that contrasts the local field, so that the projection of the total force along the direction of the force-field has opposite direction with respect to 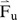. **Figure S6. Task A: typical trajectory and force profiles during corrective motions.** The figure show a transient motion between two stabilization phases for the subject 6 (S6) for the first session in the case of the SSS (top panel) and the PSS (bottom panel). Left graphs represent the tool-tip trajectory (in black); red circles represent the initial (top circle) and final (bottom circle) target areas; grey arrows represent the saddle-like force-field. The top-right graphs represent the forces imposed by the hands: the right hand is in black, the left hand in grey. The bottom-right graphs depict the resultant of the forces acting on the virtual mass. In every graph, the light blue portions of the signals correspond to corrective motions that contrasts the local field, so that the projection of the total force along the direction of the force-field has opposite direction with respect to 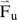. **Figure S7. Task A: typical trajectory and force profiles during corrective motions.** The figure show a transient motion between two stabilization phases for the subject 7 (S7) for the first session in the case of the SSS (top panel) and the PSS (bottom panel). Left graphs represent the tool-tip trajectory (in black); red circles represent the initial (top circle) and final (bottom circle) target areas; grey arrows represent the saddle-like force-field. The top-right graphs represent the forces imposed by the hands: the right hand is in black, the left hand in grey. The bottom-right graphs depict the resultant of the forces acting on the virtual mass. In every graph, the light blue portions of the signals correspond to corrective motions that contrasts the local field, so that the projection of the total force along the direction of the force-field has opposite direction with respect to 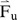. **Figure S8. Task A: typical trajectory and force profiles during corrective motions.** The figure show a transient motion between two stabilization phases for the subject 8 (S8) for the first session in the case of the SSS (top panel) and the PSS (bottom panel). Left graphs represent the tool-tip trajectory (in black); red circles represent the initial (top circle) and final (bottom circle) target areas; grey arrows represent the saddle-like force-field. The top-right graphs represent the forces imposed by the hands: the right hand is in black, the left hand in grey. The bottom-right graphs depict the resultant of the forces acting on the virtual mass. In every graph, the light blue portions of the signals correspond to corrective motions that contrasts the local field, so that the projection of the total force along the direction of the force-field has opposite direction with respect to 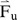. **Figure S9. Task A: typical trajectory and force profiles during corrective motions.** The figure show a transient motion between two stabilization phases for the subject 1 (S1) for the last session in the case of the SSS (top panel) and the PSS (bottom panel). Left graphs represent the tool-tip trajectory (in black); red circles represent the initial (top circle) and final (bottom circle) target areas; grey arrows represent the saddle-like force-field. The top-right graphs represent the forces imposed by the hands: the right hand is in black, the left hand in grey. The bottom-right graphs depict the resultant of the forces acting on the virtual mass. In every graph, the light blue portions of the signals correspond to corrective motions that contrasts the local field, so that the projection of the total force along the direction of the force-field has opposite direction with respect to 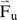. **Figure S10. Task A: typical trajectory and force profiles during corrective motions.** The figure show a transient motion between two stabilization phases for the subject 2 (S2) for the last session in the case of the SSS (top panel) and the PSS (bottom panel). Left graphs represent the tool-tip trajectory (in black); red circles represent the initial (top circle) and final (bottom circle) target areas; grey arrows represent the saddle-like force-field. The top-right graphs represent the forces imposed by the hands: the right hand is in black, the left hand in grey. The bottom-right graphs depict the resultant of the forces acting on the virtual mass. In every graph, the light blue portions of the signals correspond to corrective motions that contrasts the local field, so that the projection of the total force along the direction of the force-field has opposite direction with respect to 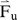. **Figure S11. Task A: typical trajectory and force profiles during corrective motions.** The figure show a transient motion between two stabilization phases for the subject 3 (S3) for the last session in the case of the SSS (top panel) and the PSS (bottom panel). Left graphs represent the tool-tip trajectory (in black); red circles represent the initial (top circle) and final (bottom circle) target areas; grey arrows represent the saddle-like force-field. The top-right graphs represent the forces imposed by the hands: the right hand is in black, the left hand in grey. The bottom-right graphs depict the resultant of the forces acting on the virtual mass. In every graph, the light blue portions of the signals correspond to corrective motions that contrasts the local field, so that the projection of the total force along the direction of the force-field has opposite direction with respect to 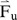. **Figure S12. Task A: typical trajectory and force profiles during corrective motions.** The figure show a transient motion between two stabilization phases for the subject 4 (S4) for the last session in the case of the SSS (top panel) and the PSS (bottom panel). Left graphs represent the tool-tip trajectory (in black); red circles represent the initial (top circle) and final (bottom circle) target areas; grey arrows represent the saddle-like force-field. The top-right graphs represent the forces imposed by the hands: the right hand is in black, the left hand in grey. The bottom-right graphs depict the resultant of the forces acting on the virtual mass. In every graph, the light blue portions of the signals correspond to corrective motions that contrasts the local field, so that the projection of the total force along the direction of the force-field has opposite direction with respect to 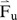. **Figure S13. Task A: typical trajectory and force profiles during corrective motions.** The figure show a transient motion between two stabilization phases for the subject 5 (S5) for the last session in the case of the SSS (top panel) and the PSS (bottom panel). Left graphs represent the tool-tip trajectory (in black); red circles represent the initial (top circle) and final (bottom circle) target areas; grey arrows represent the saddle-like force-field. The top-right graphs represent the forces imposed by the hands: the right hand is in black, the left hand in grey. The bottom-right graphs depict the resultant of the forces acting on the virtual mass. In every graph, the light blue portions of the signals correspond to corrective motions that contrasts the local field, so that the projection of the total force along the direction of the force-field has opposite direction with respect to 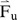. **Figure S14. Task A: typical trajectory and force profiles during corrective motions.** The figure show a transient motion between two stabilization phases for the subject 6 (S6) for the last session in the case of the SSS (top panel) and the PSS (bottom panel). Left graphs represent the tool-tip trajectory (in black); red circles represent the initial (top circle) and final (bottom circle) target areas; grey arrows represent the saddle-like force-field. The top-right graphs represent the forces imposed by the hands: the right hand is in black, the left hand in grey. The bottom-right graphs depict the resultant of the forces acting on the virtual mass. In every graph, the light blue portions of the signals correspond to corrective motions that contrasts the local field, so that the projection of the total force along the direction of the force-field has opposite direction with respect to 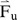. **Figure S15. Task A: typical trajectory and force profiles during corrective motions.** The figure show a transient motion between two stabilization phases for the subject 7 (S7) for the last session in the case of the SSS (top panel) and the PSS (bottom panel). Left graphs represent the tool-tip trajectory (in black); red circles represent the initial (top circle) and final (bottom circle) target areas; grey arrows represent the saddle-like force-field. The top-right graphs represent the forces imposed by the hands: the right hand is in black, the left hand in grey. The bottom-right graphs depict the resultant of the forces acting on the virtual mass. In every graph, the light blue portions of the signals correspond to corrective motions that contrasts the local field, so that the projection of the total force along the direction of the force-field has opposite direction with respect to 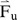. **Figure S16. Task A: typical trajectory and force profiles during corrective motions.** The figure show a transient motion between two stabilization phases for the subject 8 (S8) for the last session in the case of the SSS (top panel) and the PSS (bottom panel). Left graphs represent the tool-tip trajectory (in black); red circles represent the initial (top circle) and final (bottom circle) target areas; grey arrows represent the saddle-like force-field. The top-right graphs represent the forces imposed by the hands: the right hand is in black, the left hand in grey. The bottom-right graphs depict the resultant of the forces acting on the virtual mass. In every graph, the light blue portions of the signals correspond to corrective motions that contrasts the local field, so that the projection of the total force along the direction of the force-field has opposite direction with respect to 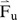.(ZIP)
