# Supplementary material for: Strategy Switching in the Stabilization of Unstable Dynamics
Source: PLoS One. 2014 Jun 12;9(6):e99087. doi: 10.1371/journal.pone.0099087 (PMC4055681; doi:10.1371/journal.pone.0099087)
Supplement: File S1 — Combined file of supporting tables and figures. Table S1. Performance Indicators in Task A. Figure S1. Task A: typical trajectory and force profiles during corrective motions. The figure show a transient motion between two stabilization phases for the subject 1 (S1) for the first session in the case of the SSS (top panel) and the PSS (bottom panel). Left graphs represent the tool-tip trajectory (in black); red circles represent the initial (top circle) and final (bottom circle) target areas; grey arrows represent the saddle-like force-field. The top-right graphs represent the forces imposed by the hands: the right hand is in black, the left hand in grey. The bottom-right graphs depict the resultant of the forces acting on the virtual mass. In every graph, the light blue portions of the signals correspond to corrective motions that contrasts the local field, so that the projection of the total force along the direction of the force-field has opposite direction with respect to . Figure S2. Task A: typical trajectory and force profiles during corrective motions. The figure show a transient motion between two stabilization phases for the subject 2 (S2) for the first session in the case of the SSS (top panel) and the PSS (bottom panel). Left graphs represent the tool-tip trajectory (in black); red circles represent the initial (top circle) and final (bottom circle) target areas; grey arrows represent the saddle-like force-field. The top-right graphs represent the forces imposed by the hands: the right hand is in black, the left hand in grey. The bottom-right graphs depict the resultant of the forces acting on the virtual mass. In every graph, the light blue portions of the signals correspond to corrective motions that contrasts the local field, so that the projection of the total force along the direction of the force-field has opposite direction with respect to . Figure S3. Task A: typical trajectory and force profiles during corrective motions. The figure show a transi [file pone.0099087.s001.zip › TableS1.doc]

Table S1. Performance Indicators in Task A

| Subject | Strategy | TS | SSI | SOI | E | TT | BSI | MVP |
| --- | --- | --- | --- | --- | --- | --- | --- | --- |
| S1 | SSS | PRE | 1.39±0.03 | 0.99±0.01 | 53.8±2.3 | 4.64±0.66 | 17.61±0.53 | 1.74±0.19 |
|  |  | POST | 1.32±0.02 | 0.99±0.01 | 48.4±1.5 | 1.88±0.22 | 16.58±0.42 | 1.52±0.19 |
|  | PSS | PRE | 0.95±0.03 | 0.92±0.03 | 23.6±1.7 | 42.06±9.79 | 1.77±0.32 | 1.41±0.15 |
|  |  | POST | 0.83±0.02 | 0.85±0.04 | 17.0±1.4 | 7.06±1.07 | 0.83±0.04 | 1.48±0.11 |
| S2 | SSS | PRE | 1.27±0.01 | 0.99±0.01 | 44.4±1.2 | 4.41±0.61 | 15.33±0.31 | 1.67±0.11 |
|  |  | POST | 1.35±0.02 | 0.99±0.01 | 49.5±1.3 | 1.13±0.16 | 16.96±0.37 | 1.68±0.19 |
|  | PSS | PRE | 0.95±0.03 | 0.84±0.08 | 24.2±1.9 | 32.09±8.8 | 0.65±0.14 | 1.56±0.18 |
|  |  | POST | 0.76±0.02 | 0.75±0.07 | 14.1±1.3 | 1.71±0.20 | 0.31±0.03 | 1.38±0.09 |
| S3 | SSS | PRE | 1.25±0.02 | 0.99±0.01 | 42.6±1.7 | 7.43±1.27 | 14.66±0.45 | 1.47±0.10 |
|  |  | POST | 1.06±0.01 | 0.99±0.01 | 29.6±0.9 | 2.06±0.54 | 11.12±0.24 | 1.18±0.09 |
|  | PSS | PRE | 0.96±0.03 | 0.87±0.06 | 24.7±1.9 | 37.85±6.37 | 0.95±0.07 | 1.10±0.08 |
|  |  | POST | 0.79±0.02 | 0.81±0.07 | 14.9±1.4 | 4.04±1.06 | 0.67±0.05 | 1.34±0.13 |
| S4 | SSS | PRE | 1.34±0.01 | 0.99±0.01 | 48.4±0.8 | 3.66±0.43 | 16.76±0.23 | 1.25±0.08 |
|  |  | POST | 1.16±0.02 | 0.99±0.01 | 35.8±1.4 | 2.05±0.41 | 13.13±0.40 | 1.35±0.12 |
|  | PSS | PRE | 0.81±0.03 | 0.58±0.10 | 16.8±1.6 | 10.88±1.06 | 1.20±0.09 | 1.30±0.14 |
|  |  | POST | 0.76±0.02 | 0.62±0.10 | 13.9±1.2 | 6.32±0.35 | 1.07±0.08 | 1.25±0.09 |
| S5 | SSS | PRE | 1.45±0.05 | 0.99±0.01 | 61.9±4.5 | 6.68±1.03 | 18.84±0.96 | 1.60±0.16 |
|  |  | POST | 1.16±0.02 | 0.99±0.01 | 36.7±1.6 | 1.55±0.20 | 13.33±0.50 | 1.19±0.11 |
|  | PSS | PRE | 0.99±0.03 | 0.85±0.07 | 26.2±1.7 | 38.89±6.99 | 1.12±0.12 | 1.11±0.12 |
|  |  | POST | 0.76±0.02 | 0.73±0.07 | 13.5±1.2 | 5.75±0.18 | 0.90±0.06 | 1.11±0.17 |
| S6 | SSS | PRE | 1.88±0.08 | 0.97±0.01 | 112.5±7.9 | 21.59±7.29 | 28.44±1.42 | 1.73±0.17 |
|  |  | POST | 1.01±0.03 | 0.92±0.05 | 27.3±1.9 | 6.77±1.09 | 9.04±0.82 | 1.40±0.12 |
|  | PSS | PRE | 0.95±0.02 | 0.84±0.09 | 29.8±1.8 | 116.89±30.29 | 1.09±0.09 | 1.77±0.16 |
|  |  | POST | 0.81±0.02 | 0.76±0.07 | 16.0±1.2 | 6.77±0.98 | 0.70±0.06 | 1.34±0.14 |
| S7 | SSS | PRE | 2.12±0.08 | 0.97±0.01 | 138.3±10.4 | 3.29±0.62 | 33.09±0.02 | 1.65±0.16 |
|  |  | POST | 1.26±0.02 | 0.93±0.06 | 43.6±0.9 | 1.70±0.26 | 15.48±0.01 | 1.45±0.10 |
|  | PSS | PRE | 0.95±0.02 | 0.87±0.08 | 29.6±1.6 | 138.53±44.12 | 0.66±0.09 | 1.38±0.13 |
|  |  | POST | 0.77±0.02 | 0.80±0.07 | 14.1±1.2 | 3.51±0.60 | 0.36±0.04 | 1.44±0.11 |
| S8 | SSS | PRE | 1.77±0.11 | 0.99±0.01 | 100.2±16.9 | 4.05±0.44 | 25.41±2.18 | 1.99±0.23 |
|  |  | POST | 1.32±0.02 | 0.99±0.01 | 47.2±1.3 | 2.08±0.25 | 16.45±0.32 | 1.13±0.08 |
|  | PSS | PRE | 1.05±0.02 | 0.93±0.05 | 30.3±1.6 | 73.51±15.03 | 1.01±0.08 | 0.98±0.08 |
|  |  | POST | 0.85±0.02 | 0.84±0.06 | 18.3±1.5 | 11.02±0.73 | 0.62±0.04 | 1.23±0.12 |

Average values (with corresponding standard error) for each subject during the first (PRE) and the final (POST) target-set (TS), for the Stiffness Stabilization Strategy (SSS) and Positional Stabilization Strategy (PSS), respectively. SSI = Stiffness Size Index; SOI = Stiffness Orientation Index; E = Effort Index [N]; TT = Time to Target [s]; BSI = Bimanual Separation Index [cm]; MVP = Magnitude of the Velocity Peaks of the virtual mass [cm/s].
